# *Staphylococcus aureus* adhesion in endovascular infections is controlled by the ArlRS–MgrA signaling cascade

**DOI:** 10.1371/journal.ppat.1007800

**Published:** 2019-05-22

**Authors:** Jakub M. Kwiecinski, Heidi A. Crosby, Claire Valotteau, Joseph A. Hippensteel, Manasa K. Nayak, Anil K. Chauhan, Eric P. Schmidt, Yves F. Dufrêne, Alexander R. Horswill

**Affiliations:** 1 Department of Immunology and Microbiology, University of Colorado School of Medicine, Aurora, Colorado, United States of America; 2 Institute of Life Sciences, Université catholique de Louvain, Louvain-la-Neuve, Belgium; 3 Division of Pulmonary Sciences and Critical Care Medicine, Department of Medicine, University of Colorado School of Medicine, Aurora, Colorado, United States of America; 4 Department of Internal Medicine, University of Iowa, Iowa City, Iowa, United States of America; 5 Walloon Excellence in Life Sciences and Biotechnology (WELBIO), Wallonia, Belgium; 6 Department of Veterans Affairs Eastern Colorado Healthcare System, Denver, Colorado, United States of America; National Institutes of Health, UNITED STATES

## Abstract

*Staphylococcus aureus* is a leading cause of endovascular infections. This bacterial pathogen uses a diverse array of surface adhesins to clump in blood and adhere to vessel walls, leading to endothelial damage, development of intravascular vegetations and secondary infectious foci, and overall disease progression. In this work, we describe a novel strategy used by *S*. *aureus* to control adhesion and clumping through activity of the ArlRS two-component regulatory system, and its downstream effector MgrA. Utilizing a combination of *in vitro* cellular assays, and single-cell atomic force microscopy, we demonstrated that inactivation of this ArlRS—MgrA cascade inhibits *S*. *aureus* adhesion to a vast array of relevant host molecules (fibrinogen, fibronectin, von Willebrand factor, collagen), its clumping with fibrinogen, and its attachment to human endothelial cells and vascular structures. This impact on *S*. *aureus* adhesion was apparent in low shear environments, and in physiological levels of shear stress, as well as *in vivo* in mouse models. These effects were likely mediated by the de-repression of giant surface proteins Ebh, SraP, and SasG, caused by inactivation of the ArlRS—MgrA cascade. In our *in vitro* assays, these giant proteins collectively shielded the function of other surface adhesins and impaired their binding to cognate ligands. Finally, we demonstrated that the ArlRS—MgrA regulatory cascade is a druggable target through the identification of a small-molecule inhibitor of ArlRS signaling. Our findings suggest a novel approach for the pharmacological treatment and prevention of *S*. *aureus* endovascular infections through targeting the ArlRS—MgrA regulatory system.

## Author summary

Adhesion is central to the success of *Staphylococcus aureus* as a bacterial pathogen. We describe a novel mechanism through which *S*. *aureus* alters adhesion to ligands by regulating expression of giant inhibitory surface proteins. These giant proteins shield normal surface adhesins, preventing binding to ligands commonly found in the bloodstream and vessel walls. Using this unique regulatory scheme, *S*. *aureus* can bypass the need for individualized regulation of numerous adhesins to control overall adhesive properties. Our study establishes the importance of these giant proteins for *S*. *aureus* pathogenesis and demonstrates that a single regulatory cascade can be targeted for treating infections.

## Introduction

*Staphylococcus aureus*, a Gram-positive bacterial pathogen, is a leading cause of infections worldwide. The most severe forms are bloodstream infections, such as sepsis, endocarditis, or thrombophlebitis, characterized by their high mortality and frequent disease sequelae [1, 2]. The crucial site in endovascular infections is the endothelium—the inner lining of blood vessels, which orchestrates the host response to bloodstream pathogens. Endothelial dysfunction is central to most of the infection-caused pathology [3, 4]. Therefore, adhesion to endothelium is an essential element of *S*. *aureus* virulence during bloodstream infections. Adhesion allows *S*. *aureus* to establish stable infection foci at the vessel walls, leading to the aforementioned dysregulation of endothelial functions, vascular leak, formation of intravascular vegetations, and metastatic spread to surrounding tissues [5–7]. *S*. *aureus* survival in the bloodstream is aided by its ability to form clumps of tightly packed cells, which protects bacteria in aggregates from immune attacks, increases antibiotic resistance, and allows a coordinated secretion of virulence factors [8].

Both *S*. *aureus* clumping and endothelial adhesion depend on interaction with host molecules. Clumping is caused mainly by linking of neighboring bacteria through binding both ends of fibrinogen molecule, an abundant blood dimeric protein. *S*. *aureus* binds fibrinogen with its surface adhesins, including the clumping factors (ClfA, ClfB) or, to a lesser extent, fibronectin binding proteins (FnbpA, FnbpB) [8]. Clumping is also likely a prerequisite for the induction of coagulation and formation of fibrin-coated *S*. *aureus* aggregates, which are essential for pathogenicity [9]. Adhesion of *S*. *aureus* to endothelium is a multifactorial process, and various host ligands in vessel walls may act as anchoring points. Von Willebrand factor (vWF), present on activated endothelium, is bound by staphylococcal vWF-binding protein (vWbp), or to a lesser extent by protein A [10–12]. In addition to vWbp and protein A, ClfA, FnbpA, FnbpB, and teichoic acid can also mediate the direct adhesion of *S*. *aureus* to endothelium, though the ligands and mechanisms of this adhesion are not entirely clear [11, 13–19]. At the sites where endothelial layer is already damaged, underlying collagen of the basal membrane is exposed, to which *S*. *aureus* binds either directly with the collagen adhesin (Cna), or indirectly with vWF bridging between collagen and bacteria [10, 20]. At the sites of endothelial inflammation or damage, clots and micro-clots composed of fibrin(ogen), fibronectin and platelets appear, providing additional binding sites for *S*. *aureus* adhesins [21].

Animal experiments with *S*. *aureus* mutants deficient in the ability to clump, coagulate, or adhere, demonstrate that these properties are essential for pathogenicity in sepsis and endocarditis [8, 9, 17, 22–26]. The complicated interaction of individual *S*. *aureus* surface proteins with host ligands, and their extensive functional redundancy, however, pose a challenge for identification of the optimal treatment target. Therefore, targeting entire regulatory pathways instead of single adhesins might hold the best therapeutic promises.

Despite the importance of clumping and adhesion, little is known about the regulation of these processes during endovascular infection, and contradictory observations have been reported regarding well-known global regulatory systems Agr and SarA [27–31]. Recently, a new regulatory cascade of ArlRS–MgrA has been identified [22, 32] that consists of a two-component regulatory system ArlRS (a transmembrane sensory kinase ArlS and the response regulator ArlR), which in turn drives the expression of MgrA, a cytoplasmic DNA-binding transcription regulator. MgrA acts as a final effector and controls the gene expression pattern [22]. Inactivation of this cascade through deletion of *arlRS* or *mgrA* blocks *S*. *aureus* clumping in plasma [22, 23]. This phenotype was associated with upregulation of multiple surface proteins, including some well-known adhesins (FnbB and protein A), but also a subset of unusual giant surface proteins Ebh, SasG and SraP (predicted size ≈1.1, ≈0.2 and ≈0.2 MDa, respectively) [22]. Therefore, a question about the mechanism of the observed inhibition of clumping remains, leading us to hypothesize that this changed repertoire of surface proteins might also have a pronounced effect on *S*. *aureus* endothelial adhesion.

In this work, we show that the ArlRS–MgrA signaling cascade is a major regulator of *S*. *aureus* clumping and adhesion in physiologically relevant conditions, both in *in vitro* models and *in vivo*. This effect is exerted not through a direct regulation of proteins involved in clumping and adhesion, but indirectly through control of a set of giant surface-bound proteins (Ebh, SasG and SraP) that shield other surface proteins from binding their ligands. This reveals a novel mechanism regulating *S*. *aureus* adhesion, and identifies the ArlRS–MgrA cascade as a potential treatment target in endovascular infections.

## Results

### ArlRS–MgrA cascade controls adhesion to endovascular ligands

**Fig 1:**
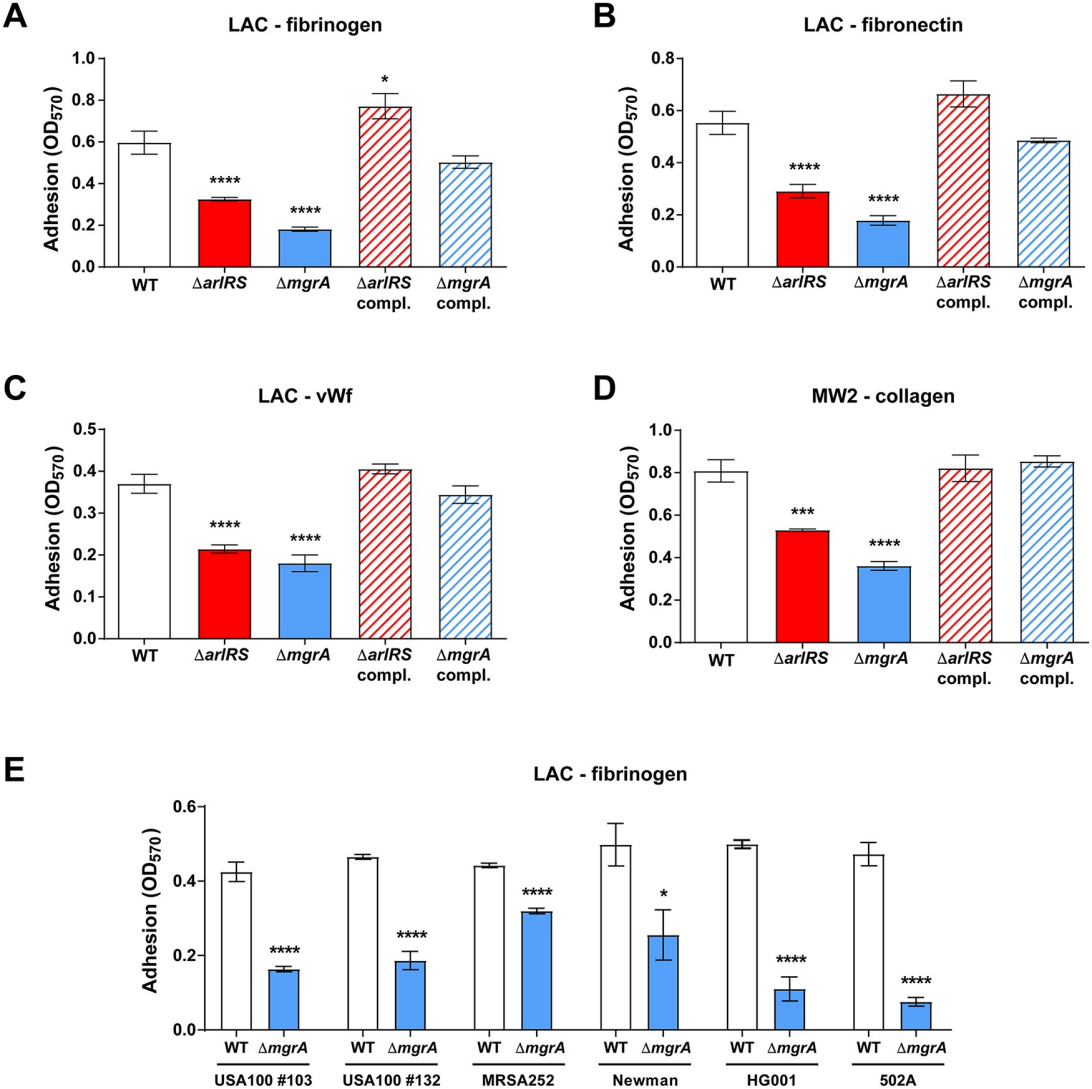
ArlRS–MgrA cascade controls adhesion to endovascular ligands in multiple *S*. *aureus* strains. Adhesion of *S*. *aureus* strains LAC (**A-C**), MW2 (**D**), and other clinical and laboratory strains of diverse lineages (**E**), and their respective mutants lacking elements of the regulatory cascade ArlRS–MgrA to fibrinogen (**A, E**), fibronectin (**B**), vWF (**C**), and collagen (**D**) was tested. Adhesions assays were performed in a static 96-well plate assay. N = 6 per group. *p<0.05, ***p<0.001, ****p<0.0001, compared to WT. Data presented as mean ± SEM.

We have previously observed that deletion of ArlRS–MgrA regulatory cascade inhibits clumping of *S*. *aureus* in the presence of fibrinogen [22, 23]. Considering the inhibitory impact on cell-to-cell interactions, we hypothesize that Δ*arlRS* and Δ*mgrA* mutants might also have defects in adhesion to surface-bound fibrinogen, and possibly also to other endovascular ligands. This was tested using *S*. *aureus* USA300 LAC strain, a prototypical community-associated MRSA strain representing the widespread USA300 clonal lineage [33]. When adhesion to a fibrinogen-coated surface was measured *in vitro*, the Δ*arlRS* and Δ*mgrA* mutant strains showed a pronounced adhesion deficit (Fig 1A). This adhesion phenotype could be reversed by complementation through chromosomal integration of the missing elements of the signaling cascade at the phage 11 integration site. In case of the complemented Δ*arlRS*, the adhesion was even slightly improved in comparison to the wild-type (WT) baseline (Fig 1A). The adhesion deficit was not limited to fibrinogen: deletion of Δ*arlRS* or Δ*mgrA* also strongly decreased binding to fibronectin and vWF, the additional ligands involved in binding to the vessel wall (Fig 1B and 1C).

The LAC strain lacks the *cna* gene encoding a collagen adhesin [33], and is accordingly incapable of adhesion to collagen. As collagen is another important ligand in the vessel wall, we tested MW2, a USA400-lineage community-acquired MRSA strain, which contains and expresses the *cna* gene [33]. The MW2 Δ*arlRS* and Δ*mgrA* deletions inhibited adhesion to collagen (Fig 1D), as well as to fibrinogen, fibronectin and vWF (S1A–S1C Fig), confirming the observations in USA300 strain. When various other *S*. *aureus* strains were screened for their adhesion to fibrinogen, the same pattern emerged. Introduction of an Δ*mgrA* mutation into MRSA clinical isolates #103 and #132 from the USA100 lineage (a lineage common in hospital-acquired bloodstream infections), USA200 lineage strain MRSA252, as well as in MSSA strains Newman, HG001, and 502a, all caused a decrease in adhesion (Fig 1E). Overall, our findings demonstrate that a deletion of the ArlRS–MgrA cascade across different *S*. *aureus* lineages has a pronounced inhibitory effect on adhesion to a diverse array of potential endovascular ligands.

### MgrA controls adhesion through regulation of giant surface proteins

**Fig 2:**
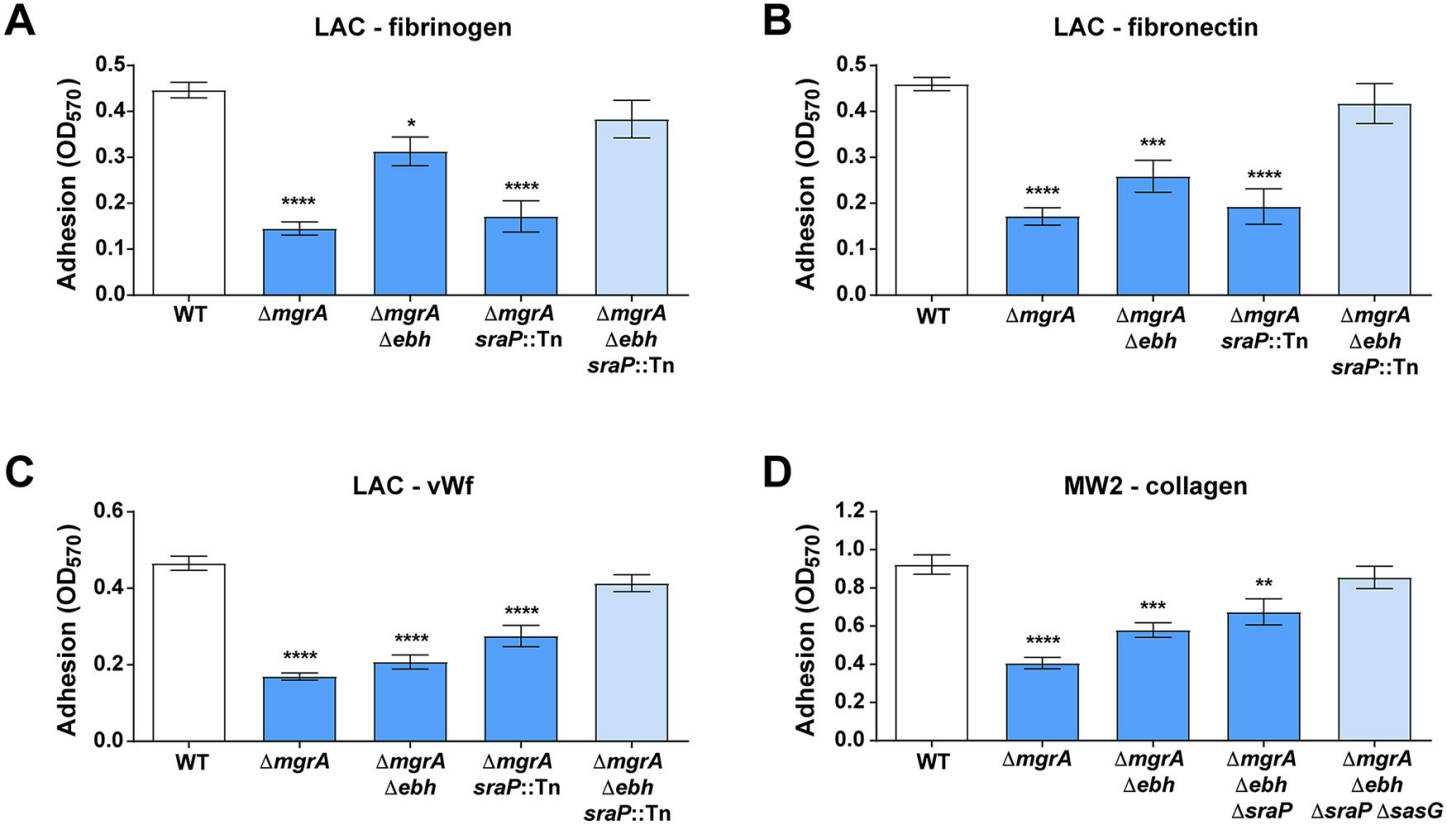
ArlRS–MgrA cascade controls adhesion through regulation of surface proteins. Adhesion of *S*. *aureus* strains LAC (**A-C**) and MW2 (**D**) with the *mgrA* deletion alone or with additional deletions of the giant surface proteins regulated by *mgrA* to fibrinogen (**A**), fibronectin (**B**), vWF (**C**), and collagen (**D**) was tested. Adhesions assays were performed in a static 96-well plate assay. N = 6 per group. *p<0.05, **p<0.01, ***p<0.001, ****p<0.0001, compared to WT. Data presented as mean ± SEM.

We previously observed that *ΔarlRS* and *ΔmgrA* mutants upregulate expression of a subset of very large surface proteins Ebh, SraP, and SasG, normally repressed by the ArlRS–MgrA cascade [22]. As USA300 strains have truncated and non-functional SasG [22], the effects of SasG are best studied in strains such as USA400 MW2. The notable upregulation of these giant surface proteins led us to hypothesize they were responsible for inhibiting adhesion in *ΔarlRS* and *ΔmgrA* strains. To test this question, we incorporated deletions of the *ebh*, *sraP*, and *sasG* genes into the *ΔmgrA* mutant background. Results confirmed that these three proteins all are jointly responsible for the observed adhesion defects (Fig 2A–2D). The relative contribution of each protein differed slightly depending on the strain and adhesion ligand. Nevertheless, only a simultaneous deletion of *ebh* and *sraP* in LAC (Fig 2A–2C), or additionally *sasG* in MW2 (Fig 2D, S1D–S1F Fig), restored the normal adhesion to various ligands in the Δ*mgrA* mutants, phenocopying the WT parent strains. Deletion of these proteins in the WT background had no effect on adhesion, in line with their low expression in the strains with a functional MgrA [22]. This parallels the previously reported role of Ebh, SraP, and SasG in inhibition of clumping [22]. Therefore, upregulation of this set of surface proteins controlled by the ArlRS–MgrA cascade is the main cause for both the lack of clumping and the inhibited adhesion.

We performed additional experiments to identify the mechanism of restored adhesion in the Δ*mgrA* mutant with Ebh, SraP, and SasG removed. Deletion of ClfA (responsible for fibrinogen binding) in LAC caused a similar decrease of fibrinogen adhesion both in the WT background and in the Δ*mgrA* Δ*ebh sraP*::Tn triple mutant (S2A Fig). In case of MW2, deletion of collagen adhesin Cna led to a similar decrease in WT background and in Δ*mgrA* mutant lacking Ebh, SraP, and SasG (S2B Fig). These observations suggest that conventional surface adhesins are still present on surface of the ArlRS–MgrA cascade mutants, and that when the giant surface inhibitory proteins are removed, conventional adhesins function normally, as in the WT strains. The presence of conventional adhesins ClfA and Cna on cell surface was further confirmed by their detection in western blots of the cell wall fraction of the *S*. *aureus* cells, and in their direct detection on cell surfaces with immunofluorescence microscopy, when characteristic surface “doughnut” staining pattern was observed (S2C–S2F Fig). Therefore, Ebh, SraP and SasG probably exert their anti-adhesive and anti-clumping effect through blocking or masking the activity of the neighboring conventional surface adhesins.

### Giant surface proteins inhibit adhesion and clumping in a dose-, location-, and size-dependent manner

Surface charge and hydrophobicity affect adhesive properties of microorganisms. However, only a modest increase in surface hydrophobicity was observed in LAC Δ*arlRS* and Δ*mgrA* mutants. This increase persisted in the Δ*mgrA* strain with deleted giant surface inhibitory proteins (S3A Fig), which otherwise phenocopies the WT strain in respect to clumping and adhesion (Fig 2A–2C), demonstrating that the changed hydrophobicity does not correlate with changes in clumping and adhesion. No significant changes in the relative negative charge on cell surface were noted (S3B Fig). These findings support our hypothesis that the giant surface inhibitory proteins exert their effect by specifically interfering with the conventional adhesins, and not through non-specific changes to the overall cell surface hydrophobicity or charge.

**Fig 3:**
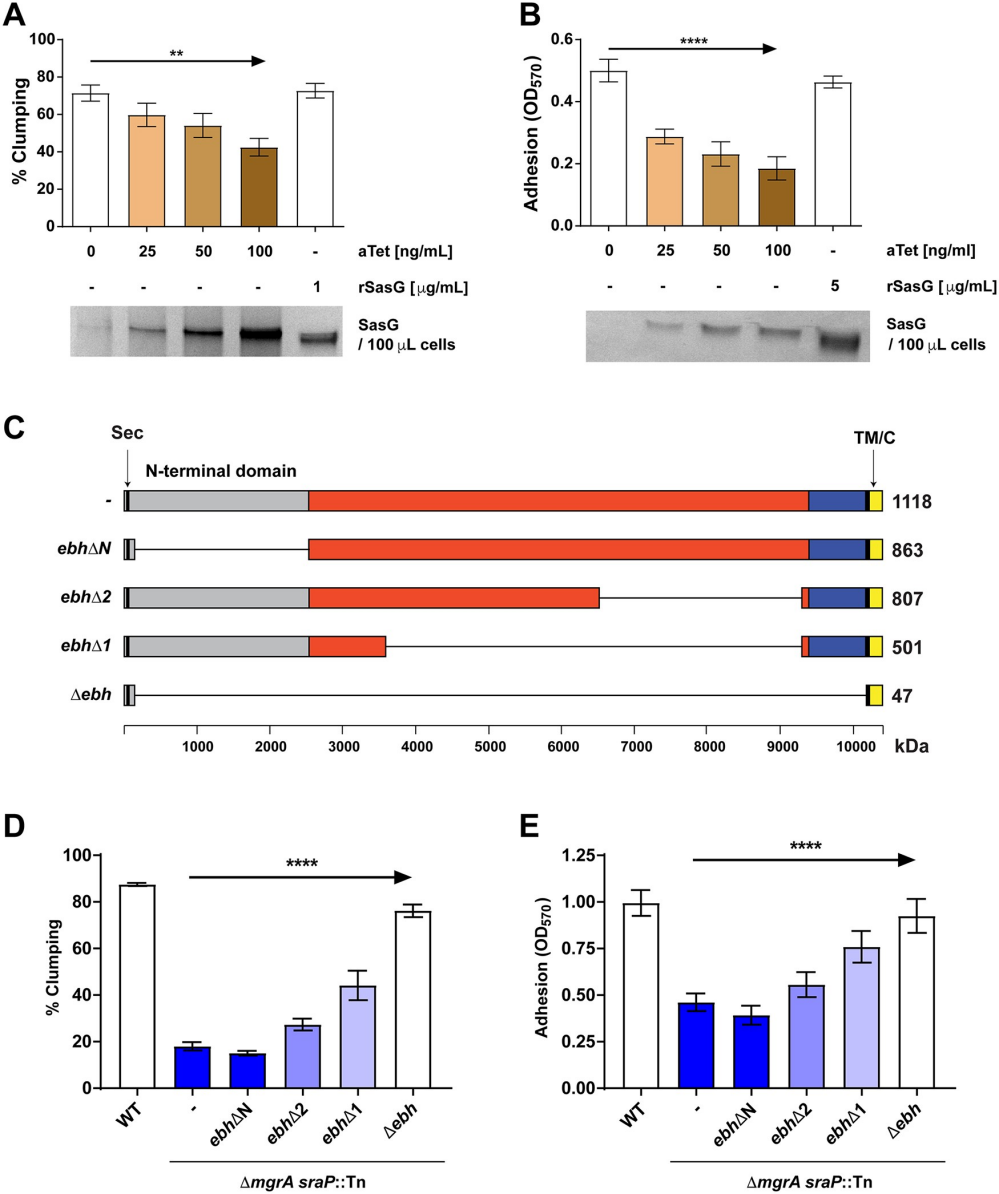
Giant surface proteins inhibit adhesion and clumping in a dose-, location-, and size-dependent manner. Clumping (**A**) and adhesion to fibrinogen in 96-well plate assay (**B**) of SasG-expressing *S*. *aureus* LAC was studied. Expression of surface-displayed SasG from Tet-inducible expression vectors pRMC2-SasG (**A**) and pALC2073-SasG (**B**) was induced by addition of anhydrotetracycline (aTet) to the growth medium. Additionally, soluble rSasG was added to some bacterial suspensions. Amount of SasG expressed by *S*. *aureus* after induction was measured by SDS-PAGE, stained with silver (**A**) or Coomassie stain (**B**), and representative images out of two independent experiments are shown. A series of chromosomal deletions in *ebh* was constructed, resulting in the expression of progressively shorter Ebh proteins in the LAC Δ*mgrA sraP*::Tn background (**C**); schemes of predicted Ebh truncated proteins are shown, with secretion signal (sec) and transmembrane and cytoplasmic domains (TM/C) highlighted. Truncation-harboring strains were tested for clumping (**D**) and fibrinogen adhesion (**E**). N = 6 (**A-B**) or 9 (**D-E**) per group. **p<0.01, ****p<0.0001. Data presented as mean ± SEM.

To further investigate the anti-adhesive phenotypes, the SasG was expressed in the LAC strain using a tetracycline-inducible promoter. As the amount of the expressed SasG increased (following increased doses of added anhydrotetracycline to the strain carrying tet-inducible pRMC2-SasG), the level of clumping decreased (Fig 3A). The same effect was observed for adhesion to fibrinogen, though the degree of inhibition was relatively small, demonstrating that inhibition of adhesion needs higher level of SasG expression than inhibition of clumping. When pALC2073-SasG, a tet-inducible relative of pRMC2 allowing for higher expression levels, was used, a clear dose-dependent inhibition of adhesion by SasG was demonstrated (Fig 3B). Further, the addition of anhydrotetracycline had no effect on the strains carrying empty expression vectors (S4A and S4B Fig), confirming that it was specifically the induced SasG, not the antibiotic, that changed the phenotype. Together, this showed that the amount of giant surface proteins correlates with the anti-adhesive and anti-clumping properties. Addition of a soluble, recombinant SasG to the assay, at an amount comparable to or even exceeding the one induced on the *S*. *aureus* surface (1–5 μg/ml), produced no observable effect (Fig 3A and 3B). This demonstrated that for their inhibitory activity, the giant proteins have to be present directly on the cell surface, possibly interacting with the neighboring conventional adhesins.

In addition to the dose- and localization-dependence, the observed inhibitory effect was also dependent on the size of the inhibitory proteins. A series of truncations were introduced in *ebh* in the chromosome of LAC (in the Δ*mgrA sraP*::Tn background), preserving the Ebh secretion signal and transmembrane/cytoplasmic domains responsible for protein anchoring, resulting in a series of progressively shorter Ebh variants (Fig 3C). All of the protein variants were correctly displayed on the cell surface as evidenced by the staining of whole cells with anti-Ebh antibody, which produced a characteristic “doughnut” pattern of surface staining, and by Ebh Western analyses (S5A–S5C Fig). When properties of this mutant series were tested, there was a correlation of the phenotype with the size of the Ebh construct. As Ebh truncations reached smaller sizes, Ebh lost its capacity to inhibit clumping and aggregation (Fig 3D and 3E), with the first effects visible after ≈1.1 MDa protein was truncated to ≈0.8 MDa, and a more pronounced effect after truncation to ≈0.5 MDa.

### Expression of giant surface proteins might lead to unclumping, detachment, and dissemination

**Fig 4:**
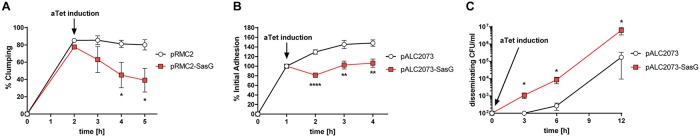
Induction of SasG expression in *S*. *aureus* leads to unclumping, detachment and dissemination. Clumping (**A**), adhesion to fibrinogen in 96-well plates (**B**), and dissemination from an infected plasma clot (**C**) of *S*. *aureus* LAC carrying Tet-inducible SasG expression vectors pRMC2-SasG (**A**) and pALC2073-SasG (**B-C**), induced by addition of BHI and 400 ng/ml anhydrotetracycline (aTet). N = 6 per group. *p<0.05, **p<0.01, ****p<0.0001. Data presented as mean ± SEM.

The observed inhibition of clumping and adhesion caused by expression of giant surface proteins raised the possibility that these might also cause detachment of individual bacteria from already established clumps or infectious foci. Indeed, when expression of *sasG* was induced in an already clumped LAC, it led to a marked destruction of existing clumps (Fig 4A). Similar, though less dramatic, effect occurred when expression of *sasG* was induced in LAC that was already adherent to fibrinogen. Expression of *sasG* caused a small degree of detachment, and prevented further accumulation of bacteria on fibrinogen-coated surface (Fig 4B). To simulate conditions occurring during dissemination from an infected vegetation in vasculature, we measured the effect of *sasG* induction on dissemination from an infected plasma clot under shear. In this setting, the strain expressing SasG disseminated from inside the infected clot to the surrounding medium quicker and to a greater degree than the strain lacking *sasG* (Fig 4C). No differences in unclumping, detachment, nor in dissemination from the clot between strains were observed in absence of induction (S6A–S6C Fig), confirming that expression of *sasG* (as opposed to the mere presence of the gene) was necessary to cause these effects. Altogether, these experiments demonstrate that regulation of giant surface proteins by the ArlRS–MgrA cascade might potentially play a role not only in prevention of clumping and attachment, but also possibly participate in regulation of *S*. *aureus* dissemination.

### Effects of adhesion regulation by the ArlRS–MgrA cascade are evident at a single-cell and single-molecule level

**Fig 5:**
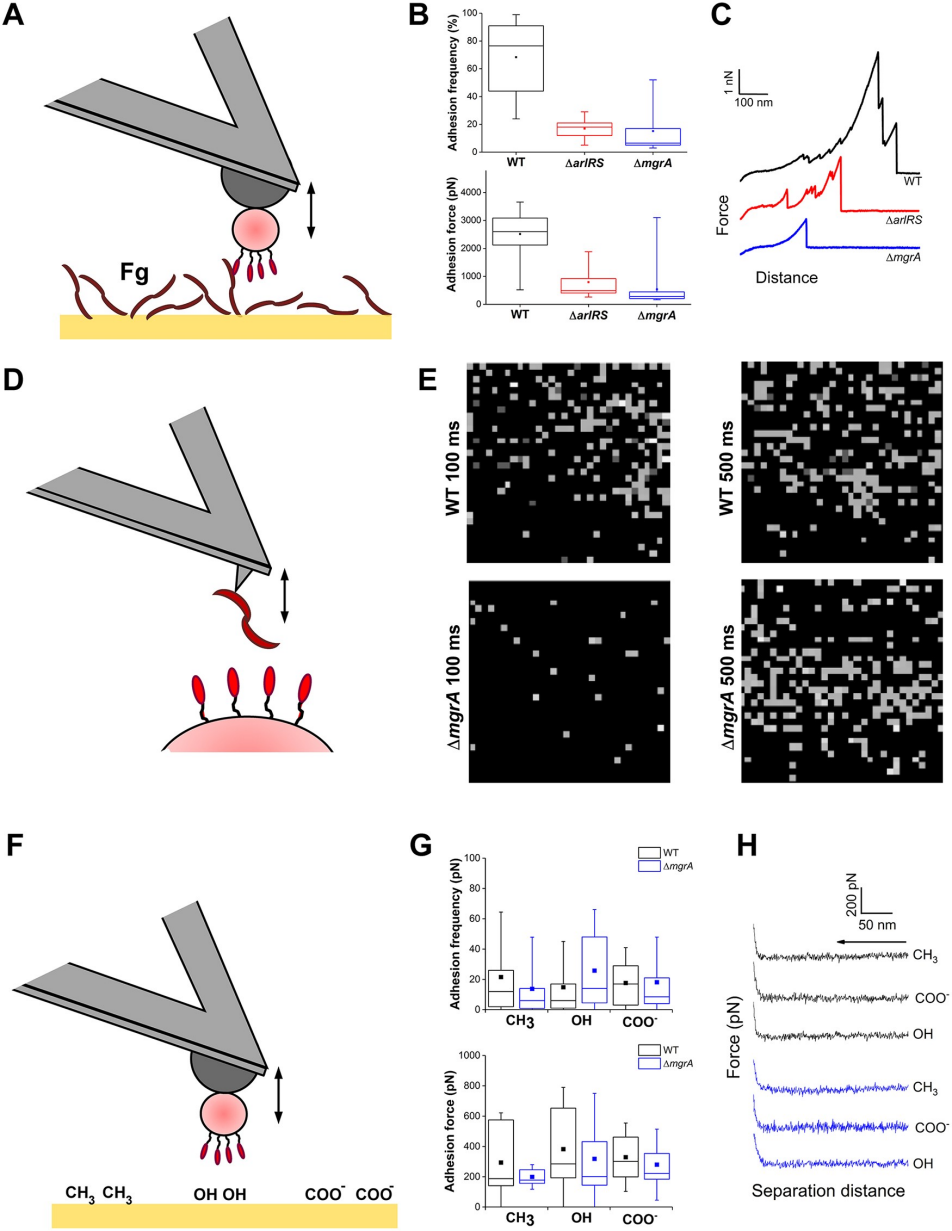
Effects of adhesion regulation by the ArlRS–MgrA cascade are evident in the single-cell and single-molecule analyses. Single-cell force spectroscopy (SCFS) was used to measure the forces between single bacteria and fibrinogen (Fg) immobilized on solid surfaces (**A**). Box-plots show the distributions of adhesion frequencies (top) and maximum adhesion forces (bottom) for the interaction, in buffer, between single cells from the WT, Δ*arlRS* or Δ*mgrA* strains respectively and Fg surfaces (**B**), n = 14 (WT), 5 (Δ*arlRS*) or 12 (Δ*mgrA*). Representative retraction force curves for this experiment are shown (**C**). Single-molecule force spectroscopy (SMFS) was employed to map the distribution of single Fg-binding proteins on living bacteria (**D**). This generated adhesion force maps (scan size: 500x500 nm^2^; color range: 3,000 pN) recorded on WT and Δ*mgrA* cells, using a contact time of 100 ms or 500 ms (**E**). We also used SCFS to quantify the forces between single bacteria and abiotic surfaces (**F**). Box-plots show the distribution of adhesion frequencies (top) and maximum adhesion forces (bottom) between single bacterial cells from the WT or Δ*mgrA* strains and methyl-, hydroxyl- or carboxyl-terminated surfaces (**G**), n = 7 for each strain. Representative approach curves for the different conditions in this experiment are shown (**H**). Unless stated otherwise, all curves were obtained using an applied force of 250 pN, an approach and retraction speed of 1.0 μm.s^-1^ and a contact time of 100 ms. For all box-plots, box range = percentile 25 and 75; □ = mean; whiskers = minimum and maximum of the dataset.

Conventional assays measure the “bulk” properties of the entire *S*. *aureus* population, but do not offer insight into behavior of individual cells or the molecular forces governing adhesion. To understand the molecular events behind the non-adherent phenotype in Δ*arlRS* and Δ*mgrA* mutants, the interaction forces of single cells were studied by atomic force microscopy (AFM) [34]. Individual bacterial cells were immobilized on AFM cantilevers and used to probe the fibrinogen-covered surfaces, while measuring adhesion forces (Fig 5A). In case of the LAC WT strain, a high frequency of adhesion events was observed, with strong force peaks of 2,368 ± 946 pN (n = 4,406 adhesive curves from a total of 14 cells) being found in most of the obtained force curves (Fig 5B and 5C). These very strong adhesion forces are in the range of those measured previously with the same assay for staphylococcal adhesins [35–37]. They are consistent with the high-affinity “dock, lock and latch” and “collagen hug” ligand binding mechanisms, which involve dynamic conformational changes of the adhesins resulting in highly stabilized adhesin-ligand complexes [38, 39]. These binding forces were missing in the cells of Δ*arlRS* and Δ*mgrA* mutants, where both the frequency and the observed forces of adhesion were dramatically reduced, especially in the Δ*mgrA* strain (Fig 5B and 5C). These results provide direct evidence that fully functional fibrinogen-binding adhesins are exposed on the surface of the *S*. *aureus* WT cells, and that their “dock, lock and latch” binding activity is strongly inhibited in the Δ*arlRS* and Δ*mgrA* mutant strains.

To analyze the distribution of adhesins on the cell surface, bacteria were mapped with the AFM tips functionalized with the fibrinogen (Fig 5D). At a short interaction time (100 ms), multiple receptors interacting with the fibrinogen were detected across the surface of LAC WT, but very few receptors were accessible for fibrinogen binding on the surface of Δ*mgrA* mutant cells (Fig 5E), consistent with the whole cell data (Fig 5B and 5C). Only when the contact time was extended (500 ms), binding of fibrinogen by surface receptors on the Δ*mgrA* was detected (Fig 5E). This supports a model where adhesins are present in the mutant cells, but their interaction with ligands is decreased by shielding caused by the neighboring giant surface proteins.

The large size of the putative inhibitory proteins appearing on the surface of the Δ*arlRS* and Δ*mgrA* mutants might indicate that they act as a kind of “molecular bumper”, non-specifically preventing interaction of bacteria with surrounding surfaces. To test if such nonspecific anti-adhesive mechanisms are present in these mutants, adhesive forces between individual *S*. *aureus* cells immobilized at the AFM tip and different abiotic surfaces (hydrophobic / hydrophilic / charged—corresponding to CH_3_, OH, and COO^-^ substrate notation, respectively) were recorded (Fig 5F). Both the adhesion frequency and adhesion forces (Fig 5G) were predictably smaller than in the case of specific adhesion to fibrinogen. We saw no substantial differences in interaction with abiotic surfaces between the LAC WT cells and the Δ*mgrA* cells. The same was true when observing the approach curves (Fig 5H), indicating that there were no major differences in long-range electrostatic and steric interactions between the strains. All together, these data suggest that ArlRS–MgrA regulated factors hinder ligand adhesion. According to our other findings (e.g. Fig 2), these factors are likely the giant surface proteins under control of this regulatory system. The most probable scenario is that the inhibitory proteins appearing on the surface of the Δ*arlRS* and Δ*mgrA* mutants are shielding the neighboring adhesins through crowding, perhaps forcing them into suboptimal conformation or preventing the three-dimensional conformational changes needed for ligand binding.

### ArlRS–MgrA cascade controls adhesion under physiological conditions

**Fig 6:**
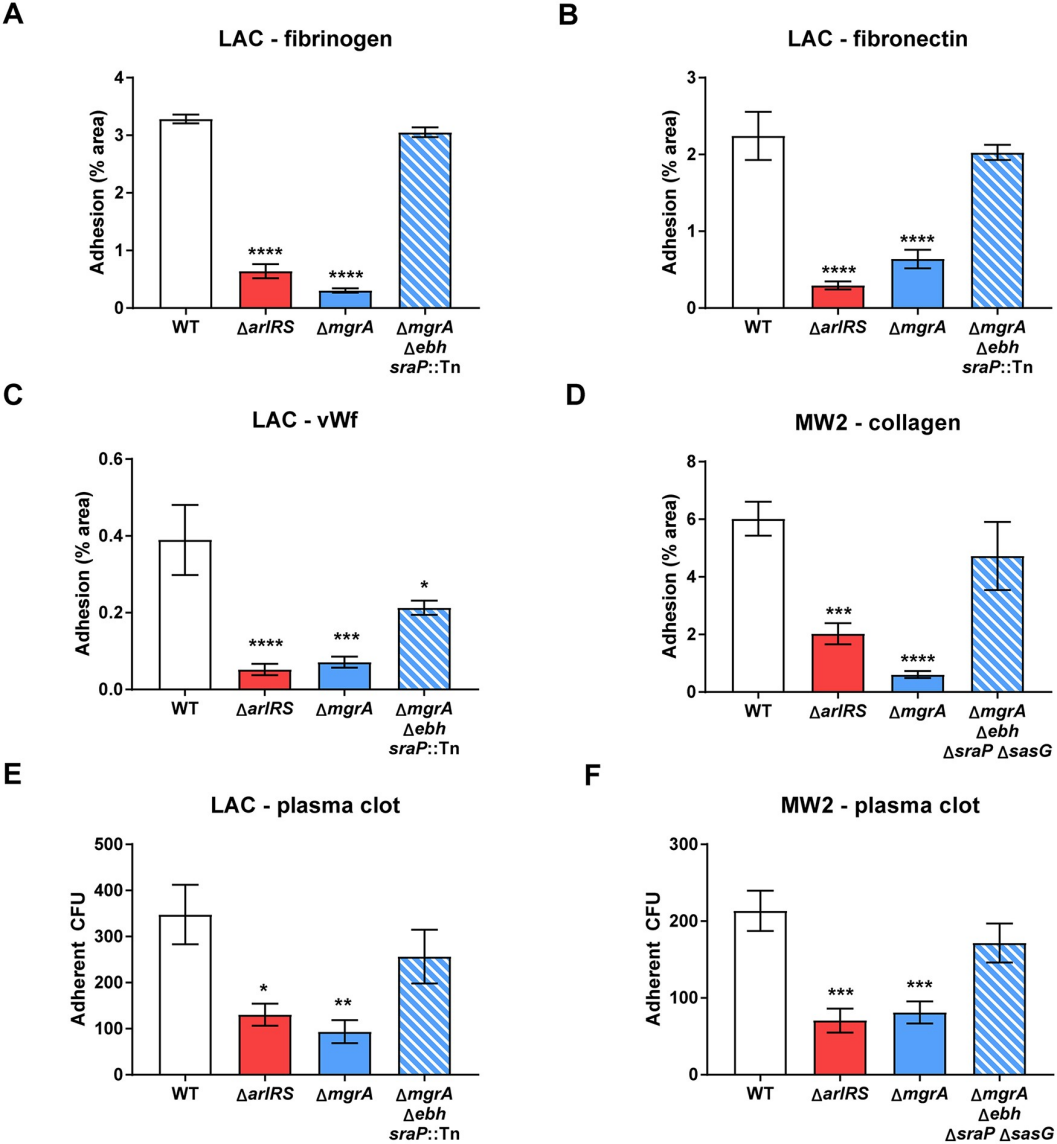
ArlRS–MgrA regulatory cascade controls adhesion under physiological conditions. Adhesion of *S*. *aureus* GFP-expressing strains LAC (**A-C**) and MW2 (**D**) with the *arlRS* or *mgrA* deletions alone or with additional deletions of the giant surface proteins regulated by the ArlRS-MgrA cascade to fibrinogen (**A**), fibronectin (**B**), vWF (**C**), and collagen (**D**) was tested in flow chamber at the physiological shear of 10 dyn/cm^2^. N = 10 per group. Additionally, adhesion of *S*. *aureus* strains LAC (**E**) and MW2 (**F**) to human plasma clot on orbital shaker (maximal shear ≈20 dyn/cm^2^) was measured, N = 6 per group. *p<0.05, **p<0.01, ***p<0.001, ****p<0.0001, compared to WT. Data presented as mean ± SEM.

Adhesion in vasculature during infection occurs in the presence of the shear force of bloodflow, with shear stress ranging from below 5 dyn/cm^2^ to around 50 dyn/cm^2^, depending on the location [40]. To translate observations from the static microwell assays into a system mimicking real-life conditions, adhesion of *S*. *aureus* was studied in the flow chambers at a shear stress of 10 dyn/cm^2^, comparable to that observed in the human body. Under these conditions, LAC readily adhered to fibrinogen, with a slightly weaker adhesion to fibronectin and collagen (the latter for MW2), and a yet weaker adhesion to vWF. Irrespective of the baseline level of the WT adhesion, the Δ*arlRS* and Δ*mgrA* mutants displayed decreased adhesion to all of the ligands (Fig 6A–6D). This adhesion deficit was much more pronounced than the one in static assays. Deletion of the giant surface protein genes *ebh*, *sraP* (and *sasG*, for the MW2 strain*)*, which are de-repressed in the Δ*arlRS* and Δ*mgrA* mutants, restored the phenotypes to the WT level (Fig 6A, 6B and 6D) or markedly increased the adhesion (Fig 6C), demonstrating that effect of the ArlRS-MgrA cascade on adhesion is mediated mainly through these giant surface proteins.

Unlike most of the *in vitro* assays, where single proteins and coated surfaces are used for the adhesion, the fibrin-embedded infectious foci at the vessel wall (as well as developing micro-thrombi at the sites of endovascular damage) are composed of multiple protein types with intricate three-dimensional architecture. This meshwork of fibrin and other proteins could trap individual bacteria in a non-specific way, promoting subsequent specific adhesion. To test how Δ*arlRS* and Δ*mgrA* mutants would behave in such conditions, their adhesion to real human plasma clots under shear was tested. In line with other results, the adhesion of the ArlRS–MgrA cascade mutants to plasma clots was significantly reduced compared to the WT strains (Fig 6E and 6F). Similar as the experiments with single purified ligands, the decreased adhesion to plasma clot in the Δ*arlRS* and Δ*mgrA* mutants could be restored to WT levels by introducing additional deletions of the giant surface proteins Ebh, SraP, and SasG (Fig 6E and 6F). This showed that also in the complex environment of a three-dimensional clot, these giant proteins are the main effectors of the observed lack of adhesion in the ArlRS-MgrA cascade mutants.

### ArlRS–MgrA cascade controls adhesion to endothelium under shear

**Fig 7:**
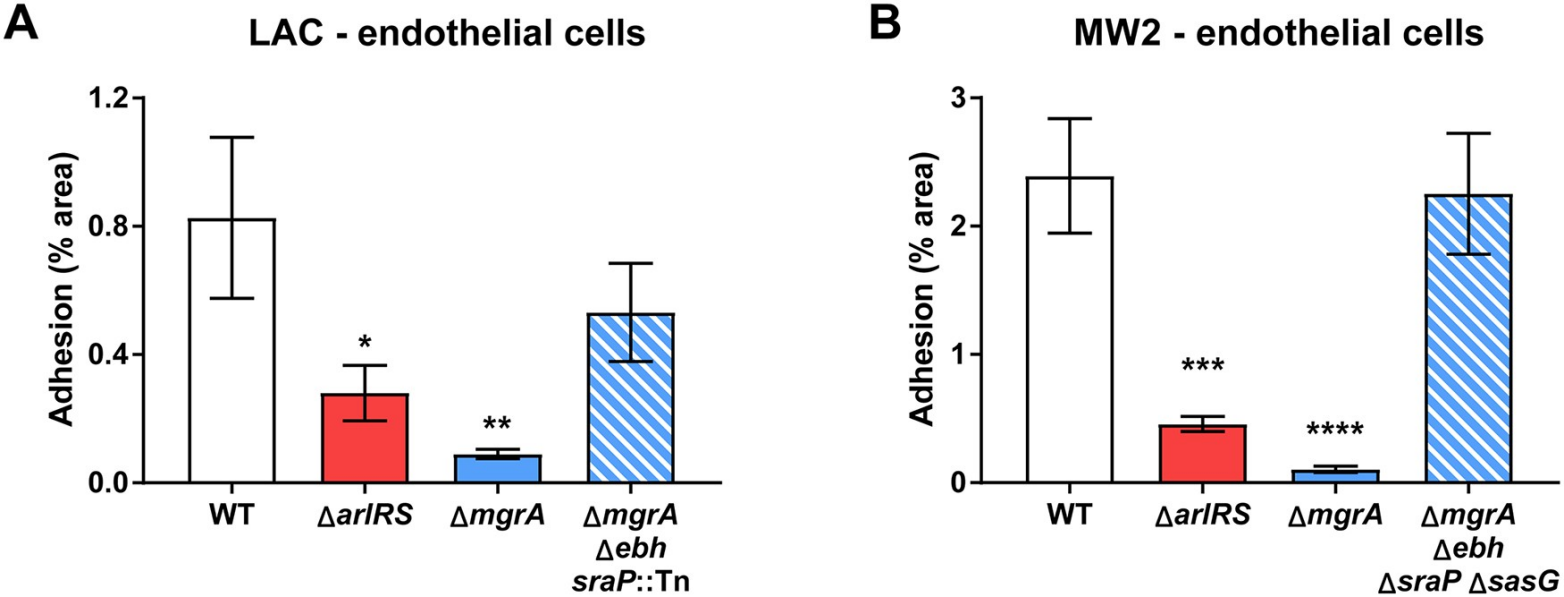
ArlRS–MgrA regulatory cascade controls adhesion to endothelium. Adhesion of *S*. *aureus* GFP-expressing strains LAC (**A**) and MW2 (**B**) with the *arlRS* or *mgrA* deletions alone or with additional deletions of the giant surface proteins regulated by the ArlRS-MgrA cascade to cultured HUVEC human endothelial cell monolayer was tested in flow chamber at the physiological shear of 10 dyn/cm^2^. N = 10 per group. *p<0.05, **p<0.01, ***p<0.001, ****p<0.0001, compared to WT. Data presented as mean ± SEM.

Many of the ligands used by *S*. *aureus* for attachment during endovascular infection would be present either in the vicinity, or directly on the endothelial cells lining the vessel. Therefore, we measured the ability of mutant strains to adhere to human endothelial cell monolayers under physiological shear of 10 dyn/cm^2^. Under these conditions, LAC bound mainly to vWF multimers displayed on the endothelial surface, and to a smaller extent directly to the endothelial cells (S7 Fig). Under these conditions, the differences between WT and mutants were clearly visible. Irrespective of the strain background, Δ*arlRS* and Δ*mgrA* mutants attached significantly less than the respective LAC and MW2 WT parents (Fig 7A and 7B). This lack of adhesion was reversed by additional deletion of genes for the giant surface proteins (Fig 7A and 7B). Thus, the ArlRS–MgrA cascade appears to be a master regulator of *S*. *aureus* adhesion to a wide variety of ligands and conditions that could occur in vasculature, and this effect appears to be mediated by the giant surface proteins Ebh, SraP, and SasG.

### ArlRS–MgrA cascade controls adhesion to vasculature *in vivo*

**Fig 8:**
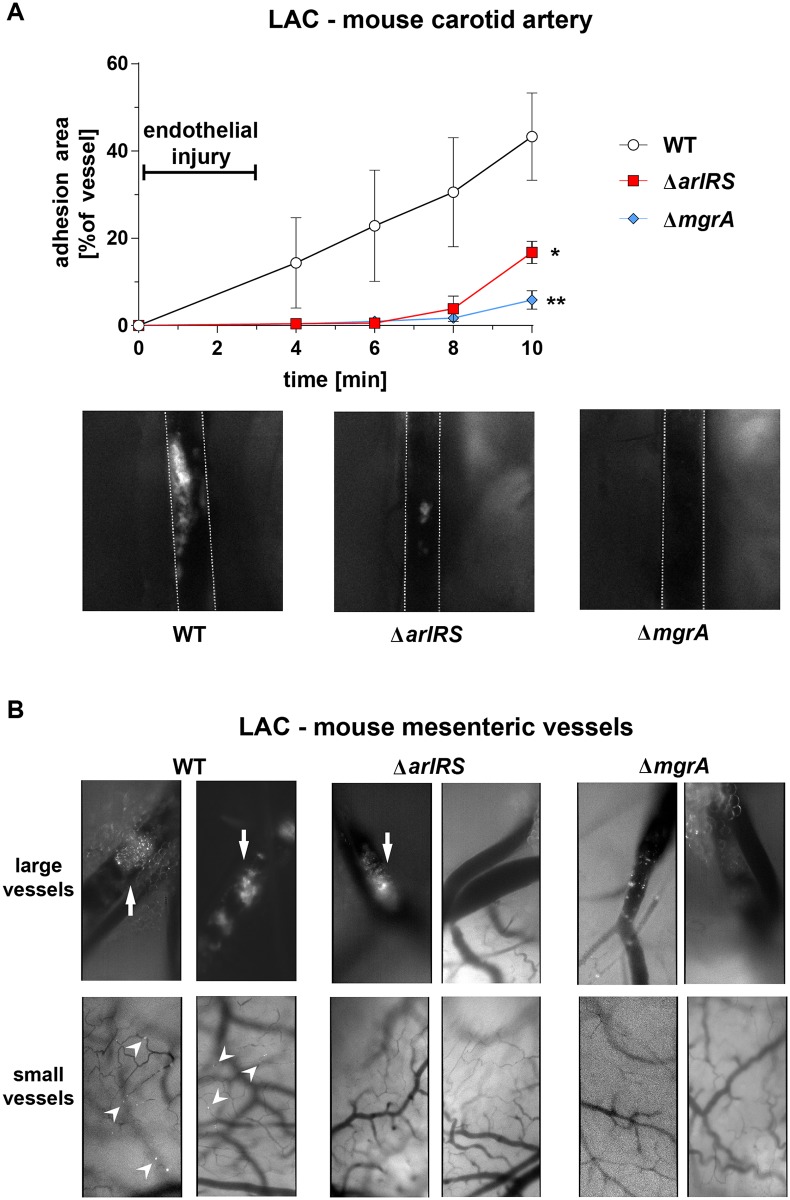
ArlRS–MgrA cascade controls adhesion to vasculature *in vivo*. Adhesion of GFP-expressing *S*. *aureus* LAC WT and its mutants to mouse carotid artery (**A**) with FeCl_3_-injured endothelium was monitored using intravital microscopy after intravenous injection of fluorescent bacteria. Representative microscopy images of artery at 8 min time-point are shown (image size: 2 mm × 2 mm; outlines of arteries marked with white lines). N = 3 per group. *p<0.05, **p<0.01, compared to WT. Data presented as mean ± SEM. Adhesion of GFP-expressing *S*. *aureus* LAC WT and its mutants to a Ca^2+^-ionophore-activated mouse mesenteric circulation (**B**) was evaluated using intravital microscopy after intravenous injection of fluorescent bacteria. Two independent experiments (2–3 mice per group) were performed, and representative images at 30 min post-injection are shown (image size: 0.5 mm × 1 mm). Large adherent clots were observed in large mesenteric vessels after injection of WT and Δ*arlRS* bacteria (arrows). Small clumps and individual adherence of WT bacteria was visible in small vasculature (arrowheads). No adherence of Δ*arlRS* and Δ*mgrA* mutants in small vessels was observed.

To investigate the behavior of *S*. *aureus* inside vasculature *in vivo*, we used intravital microscopy tracking fluorescent bacteria in a damaged mouse carotid artery. Following a chemical injury to the endothelial layer, the GFP-expressing LAC circulating in the bloodstream rapidly adhered both directly to the damaged vessel wall, and to the thrombus developing in the lumen over the injured area (Fig 8A). Strains with Δ*arlRS* and Δ*mgrA* mutations failed to adhere to the damaged murine vessel *in vivo*, consistent with our panel of *in vitr*o assays. Mutant strains began to accumulate in the artery only after a prolonged time, potentially because the developing thrombus at that time occluded most the vessel’s lumen, allowing for unspecific trapping of circulating bacteria in regions of stagnant flow. Nevertheless, even at these later time points, the strains with an inactivated ArlRS–MgrA cascade failed to accumulate to the same extent as the WT (Fig 8A). This demonstrates that hypotheses formed in our *in vitro* model systems translate to a real infectious event, and that ArlRS–MgrA is indeed crucial for regulating adhesion to vessel walls and overall behavior of *S*. *aureus* inside vasculature.

To confirm these observations in another *in vivo* setting, we used intravital microscopy to observe attachment of GFP-expressing *S*. *aureus* to mesenteric endothelium activated with a Ca^2+^-ionophore. The LAC WT strain attached to and formed large clots obstructing entire lumen of the large mesenteric vessels (Fig 8B). These large clots were observed to some degree after injection of the Δ*arlRS* mutant, but were absent after injection of the Δ*mgrA* mutant. The Δ*mgrA* didn’t attach at all, or attached to vessel walls without occluding the vessel’s lumen (Fig 8B). Differences between the strains were even more pronounced in smaller vessels. Tiny clumps and individual bacteria of WT strain attached within small vessels, while neither Δ*arlRS* nor Δ*mgrA* mutants attached to these microvessels (Fig 8B). Overall, the ArlRS–MgrA cascade played an important role in attachment of *S*. *aureus* to mouse vasculature in various *in vivo* conditions and sites.

### Inhibition of ArlRS signaling with biochanin A decreases clumping and adhesion

**Fig 9:**
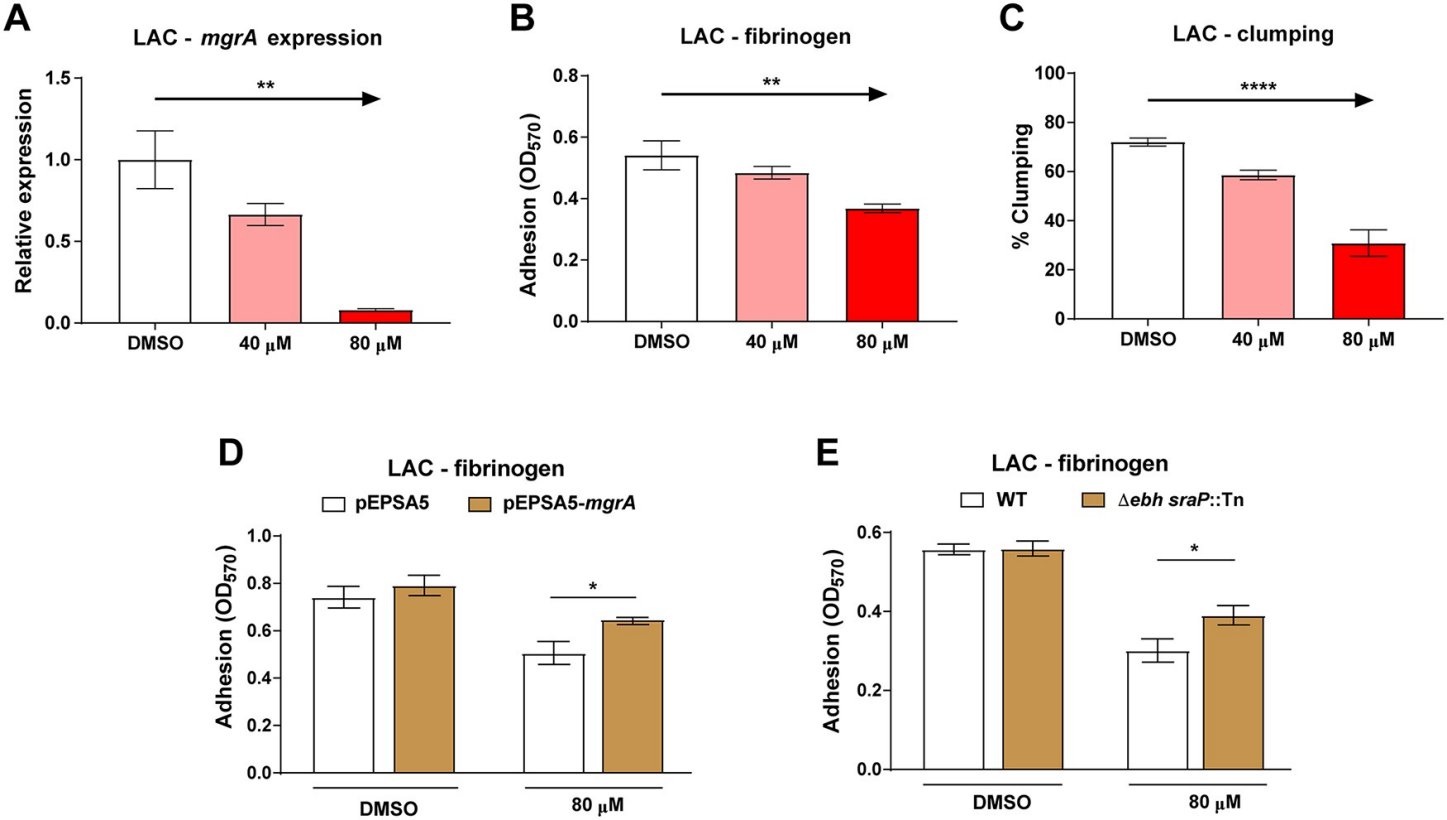
Inhibition of ArlRS signaling with biochanin A decreases clumping and adhesion. Expression of *mgrA* gene (**A,** n = 3), adhesion to fibrinogen in 96-well plates (**B,** n = 6) and clumping after 1h (**C,** n = 4) of *S*. *aureus* strain LAC grown in presence of increasing concentrations of biochanin A (or DMSO control) was tested. Adhesion to fibrinogen of *S*. *aureus* LAC carrying *mgrA* expressing complementing plasmid (**D**, n = 6) or with additional mutations of giant surface proteins (**E**, n = 6) in presence of biochanin A was also tested. *p<0.05, **p<0.01, ****p<0.0001. Data presented as mean ± SEM.

Currently, there are no known inhibitors of the ArlRS two-component system. We screened a 2320-compound small molecule library for potential new inhibitors with a previously described fluorescent reporter in which sGFP is expressed under the control of the ArlRS-dependent P2 promoter of *mgrA* [22]. This led to five potential hits (biochanin A, protoporphyrin IX, haematoxylin, chlorophyllide, and quercetin). After secondary testing of these candidates through Ebh production dot-blot assays [22, 23], and a final confirmation with qPCR measuring *mgrA* expression, biochanin A was identified as the best candidate affecting the ArlRS–MgrA cascade at doses of 40 μM and 80 μM (Fig 9A). When assayed for antimicrobial activity, biochanin A had an MIC >320 μM, approaching the solubility limit of the compound and well above the ArlRS–MgrA inhibitory doses. At sub-MIC levels, biochanin A had no impact on *S*. *aureus* growth at 40 μM, but caused a delay at 80 μM (S8 Fig). Since biochanin A can reduce *mgrA* expression at 40 μM (Fig 9A), we decided to further explore its potential as an ArlRS inhibitor. Biochanin A was able to inhibit the adhesion of *S*. *aureus* LAC to fibrinogen in a dose-dependent manner (Fig 9B), and it also triggered a significant delay in plasma-mediated clumping (Fig 9C).

To further investigate biochanin’s inhibitory effect on ArlRS signaling, and to estimate how much of the observed effect is due to its impact on the ArlRS-MgrA cascade, we performed additional experiments. We attempted to counteract the anti-adhesive effect of biochanin A by expressing *mgrA* from a plasmid, independently from the ArlRS-MgrA regulatory cascade. Complementation with additional MgrA had no effect on adhesion to fibrinogen in absence of biochanin A, but it significantly relieved part of the inhibition caused by addition of biochanin A (Fig 9D). A similar effect was observed with strains lacking giant surface proteins. Deletion of *ebh* and *sraP* had no effect on adhesion to fibrinogen in absence of biochanin A, but it led to a significantly better adhesion in the presence of this inhibitor (Fig 9E). While biochanin A may have some off-target effects on *S*. *aureus*, its anti-adhesive impact is attributable to a large extent to the inhibition of the *mgrA* expression (approximately 30–40%) and the subsequent upregulation of the giant surface proteins. Altogether, the above results suggest that ArlRS is a druggable target, and that inhibition of ArlRS could be a feasible way to intervene in *S*. *aureus* endovascular infections.

## Discussion

*S*. *aureus* bloodstream infections remain a major challenge. In the US alone, each year over 100,000 patients are hospitalized with *S*. *aureus* sepsis, and about 5,000 with *S*. *aureus* endocarditis, with incidences rising [41–43]. The mortality of these *S*. *aureus* infections remains remarkably high at 15–50% [44], with survivors suffering from debilitating disease sequelae [1]. As such, it is crucial to develop novel therapies for endovascular infections [43], and this pressing need can be met only through understanding the mechanisms underlying *S*. *aureus* virulence.

In this study, we demonstrated that *S*. *aureus* clumping in plasma and adhesion to vessel walls are controlled by a regulatory cascade composed of the ArlRS two-component system and the downstream MgrA regulator. Inactivation of this single cascade was sufficient to completely inhibit clumping and adhesion. Notably, deletion of ArlRS–MgrA components had the same phenotypic effect irrespective of the *S*. *aureus* strain background used. Previous observations already suggested involvement of ArlRS and MgrA in pathogenicity of sepsis and endocarditis [22, 23, 45–47], and recently ArlRS and MgrA were identified to be parts of a single signaling cascade [22]. However, the mechanism behind involvement of this cascade in virulence remained unclear. Our study answers this question by linking the ArlRS–MgrA cascade to the tangible elements of the disease pathogenesis: clumping and adhesion. ArlRS–MgrA regulates both of these crucial infectious processes. We demonstrate the impact of ArlRS–MgrA both at the macroscale, and at the cellular and molecular level.

Historically, bacterial adhesion was studied in static assays. Static conditions make detailed analysis of mechanisms feasible, but they differ from real-life conditions inside the host. Within the human vasculature, *S*. *aureus* attaches to vessel walls while withstanding the shearing force of the blood flow. This shear modulates conformation of bacterial surface proteins and alters their adhesive properties. Indeed, *S*. *aureus* adhesion to endothelium and endothelial ligands changes depending on the flow and shear stress [10, 30, 48–50]. In experiments where we mimicked conditions in the vasculature, the anti-adhesive effect of deletions in ArlRS–MgrA cascade become even more apparent than in static assays. Under physiological shear stress, inactivation of the ArlRS–MgrA cascade led to a complete lack of adhesion to typical endovascular ligands. The Δ*arlRS* and Δ*mgrA* mutants also showed defects in adhesion to plasma clots and endothelial layer under shear stress, representing real-life conditions where adhesion occurs in the context of complex mixtures of proteins or neighboring cells, not just single purified ligands. These behaviors of Δ*arlRS* and Δ*mgrA* mutants were replicated in two mouse models of endovascular adhesion, confirming the *in vitro* observations. These collective findings clearly demonstrate the ArlRS–MgrA signaling cascade as the major regulator of adhesion.

Noteworthy, the ArlRS–MgrA cascade does not regulate adhesion and clumping through altered expression of the well-known *S*. *aureus* adhesins previously implicated in pathogenesis (e.g. ClfA, ClfB, Coa, vWbp, FnbpA, FnbpB, protein A, or Cna) [22]. Instead, this cascade regulates expression of a subset of giant surface proteins (Ebh, SraP and SasG) that inhibit clumping and binding to a vessel wall. Of these, only SasG was previously reported to interfere with *S*. *aureus* binding to fibronectin and fibrinogen [51], but no such activity was previously known for Ebh and SraP. The most likely scenario explaining the phenotypes of defects in ArlRS–MgrA mutants is that Ebh, SraP, and SasG shield the neighboring adhesins through crowding, perhaps forcing them into suboptimal conformation or preventing the three-dimensional conformational changes needed for ligand binding. This is fully consistent with the notion that ligand binding by staphylococcal adhesins through the “dock, lock, and latch” or “collagen hug” mechanisms involves multiple structural changes [38, 39]. Inhibitory proteins will prevent optimal fitting of the interacting molecules, thus compromising the formation of stabilized complexes. This would explain both the requirement for the large size and cell-surface localization of the inhibitory proteins, their lack of unspecific effects on interaction of *S*. *aureus* with ligand-free surfaces, and their mode of action through the specific inhibition of the neighboring surface adhesins. This model is also in agreement with our single-cell force spectroscopy data. Through such crowding, a simultaneous inhibition of binding to numerous ligands can be achieved, preventing clumping and adhesion irrespective of what adhesins are present on the surface. Remarkably, the ArlRS–MgrA cascade achieves such profound phenotypic effects through regulation of only three surface proteins.

**Fig 10:**
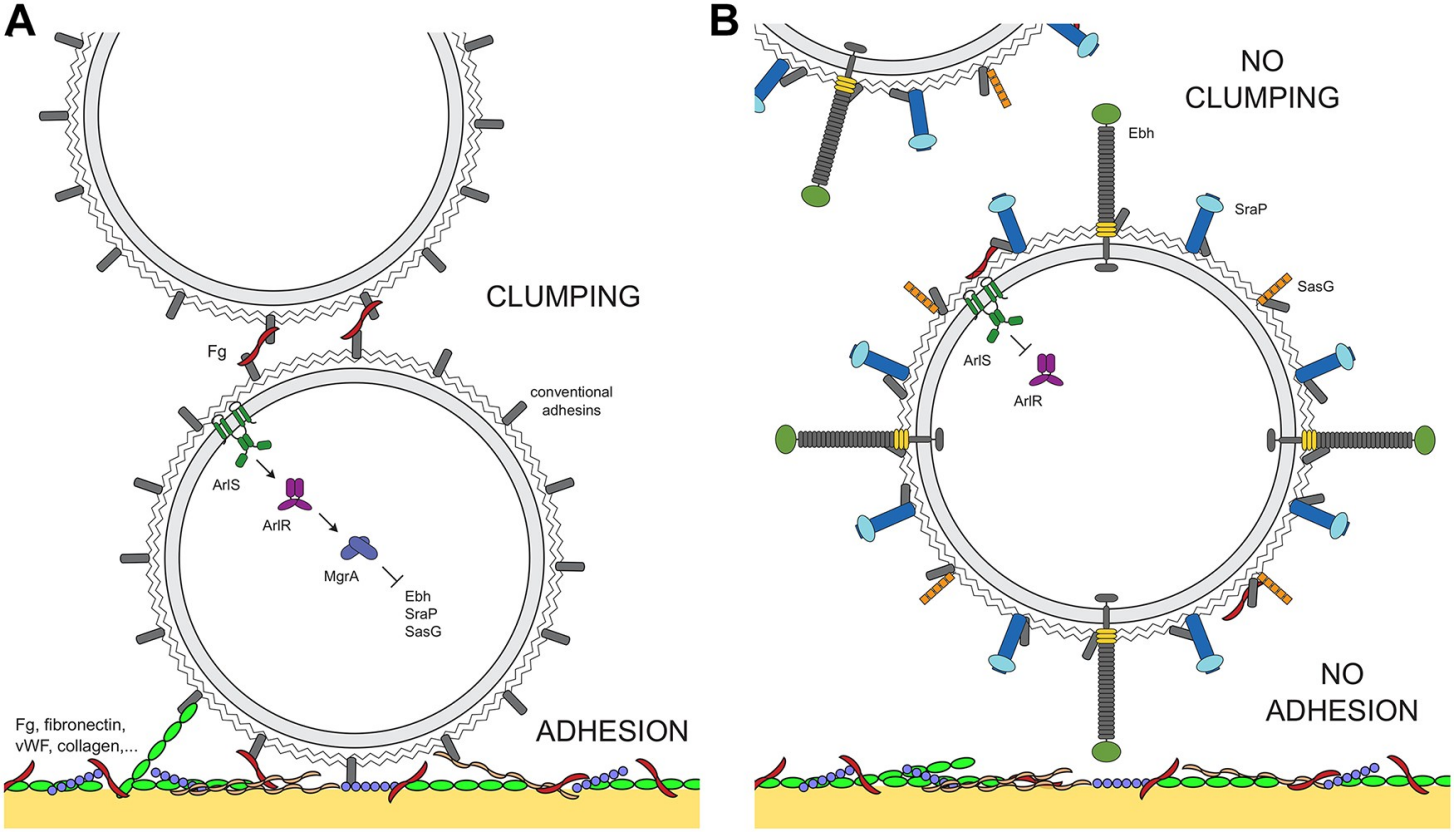
Proposed mechanism of ArlRS–MgrA cascade controlling clumping and adhesion in *S*. *aureus*. With functional ArlRS–MgrA cascade, giant surface proteins (Ebh, SraP, SasG) are repressed, and conventional surface adhesins can mediate clumping (by crosslinking cells through fibrinogen, Fg), and adhesion of *S*. *aureus* to ligands on the vessel wall (**A**). Inactivation of the ArlRS–MgrA cascade leads to de-repression of Ebh, SraP and SasG, and their appearance on *S*. *aureus* cell surface prevents neighboring adhesins from interacting with their ligands, what leads to lack of clumping and adhesion (**B**).

Overall, data collected in this study led us to construct a model linking ArlRS–MgrA signaling, clumping, and adhesion (Fig 10). When the ArlRS–MgrA cascade is active, giant surface proteins are repressed, and only conventional adhesins are present on bacterial surface. These bind fibrinogen and other common vascular ligands, leading to cells clumping and adhering to vessel walls (Fig 10A). When the ArlRS–MgrA cascade is inactive, conventional adhesins are still present, but giant proteins Ebh, Srap and SasG are de-repressed, and their appearance on bacterial surface interferes with ligand binding by the adhesins. This leads to abrogation of clumping and adhesion (Fig 10B). Unable to clump and adhere, *S*. *aureus* will be vulnerable to host attacks and will be gradually cleared from circulation. Lack of adhesion to vessel wall will prevent formation of localized infectious foci and endovascular vegetations, and will not allow for endothelial damage and dysfunction to occur, explaining recent observations of decreased killing caused by Δ*arlRS* mutant strain in cultured endothelial cells [52]. Thus, many conventional elements of disease pathogenesis in bloodstream infections will be derailed simultaneously.

Identification of the ArlRS-MgrA cascade as one of the master regulators of adhesion and clumping makes it a desirable drug target, though question remains about the role of this cascade in the normal life cycle of *S*. *aureus*. In our assays the phenotype of the Δ*mgrA* mutants was frequently more pronounced than the one of the Δ*arlRS* mutants, which results from MgrA being the direct regulator of gene expression, and from low levels of constitutive expression of *mgrA* even in the absence of ArlRS [22]. Similarly, effects of the AlrRS-MgrA regulatory cascade during naturally occurring infection might also be more gradual than in the case of Δ*mgrA* mutants, depending on the level of the ArlRS activity in response to the infection environment. Various other systems, such as *agr* and *sarA* have been implicated in regulation of staphylococcal adhesins, and ability to both display and remove adhesins from cell surface in response to environmental conditions is important for *S*. *aureus* virulence [53]. The ability of ArlRS-MgrA regulation to change the surface properties of *S*. *aureus* without a need for individual regulation of each adhesin make it especially useful for adaptation to changing environment during infection and colonization. As the signal detected by the ArlS is still unknown, we are unable to confidently identify conditions in which *S*. *aureus* uses this regulation. We speculate that the ArlRS-MgrA cascade may be employed for regulation of detachment, or for swift adaptation of bacteria to a non-vascular environment.

Our data suggested, that in addition to prevention of clumping and adhesion, the expression of Ebh, SraP and SasG may be used by *S*. *aureus* as means of unclumping and/or detachment from a surface. Considering the very strong force of ligand-adhesin bond in *S*. *aureus*, we envision that display of giant surface proteins probably does not cause immediate detachment on its own. Rather, as the clumped/adherent state is being continuously disrupted by cells divisions and/or mechanical forces of the environmental shear, the detached or newly divided cells expressing giant surface proteins will be unable to re-attach. These detached cells will continuously disseminate from the infectious foci, shifting the overall balance towards an unclumped/detached phenotype. ArlRS–MgrA may therefore be thought of as a regulatory switch between two distinct phenotypes: (i) an adhesive/clumping phenotype that is optimal for establishing infectious foci inside host’s vasculature (MgrA levels are high); and (ii) a detaching/spreading phenotype necessary for other lifestyles (MgrA levels are low). Notably, this phenomenon of detachment/spread is possibly specific only for the bloodstream, and for sites in the body where “conventional” host matrix molecules (fibrinogen, fibronectin, collagen) are abundant. At other sites, Ebh, SraP and SasG may act not as “anti-adhesins”, but rather as “alternative adhesins”. SasG is known to bind nasal epithelial cells and induce biofilm formation [22, 51, 54, 55], and SraP binds to sialylated receptors on platelets and lung epithelium [56, 57]. Binding partners are currently unknown for the N-terminal domain of Ebh, and although there are reports of an internal repeat binding fibronectin [58], it seems likely that Ebh does have another host ligand that remains to be identified. Altogether, this leads us to hypothesize that turning off the ArlRS–MgrA cascade might not only allow *S*. *aureus* to move to another body site, but also to adapt to a non-endovascular, presumably skin or mucosal environment during colonization, where the higher levels of Ebh, SraP, and SasG could facilitate adherence to relevant local ligands, and where “conventional” adhesins would not be needed. As ArlRS is also involved in regulation of *S*. *aureus* metabolism [59, 60], it is possible that ArlRS–MgrA cascade takes part in a more general adaptation to these novel niches. The ongoing effort at identification of the signals turning the cascade on and off will probably allow to elaborate this model in the future.

The important *S*. *aureus* virulence functions identified for the ArlRS–MgrA cascade suggest it has potential as a drug target. As shown herein, cascade inactivation renders *S*. *aureus* unable to clump in plasma or to adhere to vessel wall, leaving the pathogen exposed to host defenses and clearance from circulation. In this work, we identified biochanin A as a modest ArlRS inhibitor that can interfere with *S*. *aureus* adhesion and clumping, providing a proof-of-concept that ArlRS is a druggable treatment target. However, a more specific inhibitor would be necessary for a clinically meaningful outcome. These findings highlight the potential for future identification of more potent inhibitors and understanding the mechanism of their action on the ArlRS two-component system. There are reported putative MgrA inhibitors showing promising effects [8, 61, 62], though their specificity for MgrA was not established. Our studies and these previous reports underscore that the ArlRS–MgrA cascade should be considered as the potential target for treatment and prevention of endovascular infections.

In conclusion, our work identifies the ArlRS–MgrA cascade as the key regulator of *S*. *aureus* adhesion and clumping, both *in vitro* and *in vivo* in the bloodstream. This regulation is likely achieved through control of expression of the giant surface proteins Ebh, SraP, and SasG, which our data suggest interfere with conventional surface adhesins binding their host ligands, such as fibrinogen, fibronectin, vWF and collagen. This mechanism of adhesion control—not through directly regulating adhesins, but by inducing inhibitory proteins—adds a new level of sophistication to mechanisms by which *S*. *aureus* modulates its virulence. It is possible, that the ArlRS–MgrA cascade is one of the master switches controlling transition of *S*. *aureus* from one body site to another, or from colonization to infection. In order to improve our understanding of ArlRS–MgrA signaling, it will be important to determine the complete list of processes controlled by this cascade, identify the environmental cues sensed by ArlS, and uncover the ligands possibly bound by Ebh, SraP, and SasG. Finally, disruption of the ArlRS–MgrA cascade has potential as a therapeutic strategy, which could ameliorate the enormous burden currently imposed by *S*. *aureus* bloodstream infections.

## Materials and methods

### Ethics statement

The animal experiments were approved by the University of Iowa Institutional Animal Care and Use Committee, protocol number 6041727, and by the University of Colorado Institutional Animal Care and Use Committee, protocol number 00439. Both the University of Iowa and the University of Colorado are AAALAC accredited, and their centralized facilities meet and adhere to the standards in the “Guide for the Care and Use of Laboratory Animals.”

Human plasma used in clumping assays was obtained from the already existing anonymized plasma collection from healthy donors at the University of Iowa Inflammation Program, with all necessary institutional approvals. Plasma was diluted 1:1 with heparin/dextran sulfate to prevent clotting (this mixture referred to as 100% plasma). Plasma for other assays was obtained from University of Iowa DeGowin Blood Center, as an anonymized apheresis plasma anticoagulated with the acid citrate dextrose solution. No regulatory approval was needed for using this blood product.

### Bacteria and standard growth conditions

Bacterial strains and plasmids used are listed in the Table 1. Two USA100 clinical isolates from bloodstream infections in the University of Iowa Hospitals and Clinics were kindly donated by Dr. Daniel Diekema. For all experiments, *S*. *aureus* cultures were grown in a brain-heart infusion broth (BHI) at 37°C, with shaking to an OD_600_ = 1.5, washed with phosphate buffered saline (PBS), resuspended in the same volume of PBS, and directly used for assays or further adjusted to a desired concentration as needed. *Escherichia coli* cultures were grown in LB broth or agar. Antibiotics were added to media as needed: chloramphenicol (Cam), 10 μg/ml; erythromycin (Erm), 5 μg/ml; tetracycline (Tet), 1 μg/ml; ampicillin (Amp), 100 μg/ml, spectinomycin (Spec), 50 μg/ml.

**Table 1:** Strains and plasmids used in this study.

Strain / plasmid	Description	Source / reference
*E*. *coli*		
DH5α	host for plasmid construction	NEB
ER2566	overexpression strain	NEB
*S*. *aureus*		
RN4220	restriction deficient cloning host	[63]
AH1263	USA300 CA-MRSA, erm^S^ (= LAC*)	[64]
AH2146	LAC* φ11::LL29tet (= tet^R^ LAC* variant, used for experiments with tetracycline-inducible expression vectors)	this work
AH3052	LAC* Δ*spa*	[22]
AH3007	LAC* *spa*::φNΣ	[23]
AH1975	LAC* Δ*arlRS*	[23]
AH3244	LAC* Δ*arlRS* φ11::LL29tet *arlRS*	this work
AH3455	LAC* Δ*mgrA*::tetM	[22]
AH3458	LAC* Δ*mgrA*::tetM Δ*spa*	[22]
AH3488	LAC* Δ*mgrA*::tetM *spa*::φNΣ	[22]
AH3485	LAC* Δ*mgrA*::tetM φ11::LL29erm *mgrA*	[22]
AH3490	LAC* Δ*mgrA*::tetM φ11::LL29erm *mgrA* Δ*spa*	this work
AH3150	LAC* Δ*ebh*	[22]
AH3808	LAC* *sraP*::φNΣ	[22]
AH5137	LAC* Δ*ebh sraP*::φNΣ	this work
AH3481	LAC* Δ*mgrA*::tetM Δ*ebh*	[22]
AH3811	LAC* Δ*mgrA*::tetM *sraP*::φNΣ	[22]
AH3798	LAC* Δ*mgrA*::tetM *sraP*::φNΣ Δ*ebh*	[22]
AH3885	LAC* Δ*mgrA*::tetM *sraP*::φNΣ *ebh*ΔN	this work
AH3886	LAC* Δ*mgrA*::tetM *sraP*::φNΣ *ebh*Δ1	this work
AH3887	LAC* Δ*mgrA*::tetM *sraP*::φNΣ *ebh*Δ2	this work
AH3487	LAC* Δ*mgrA*::tetM Δ*ebh spa*::φNΣ	[22]
AH4341	LAC* Δ*mgrA*::tetM *ebh*ΔN *spa*::φNΣ	this work
AH4342	LAC* Δ*mgrA*::tetM *ebh*Δ1 *spa*::φNΣ	this work
AH4343	LAC* Δ*mgrA*::tetM *ebh*Δ2 *spa*::φNΣ	this work
AH4037	LAC* Δ*clfA*	this work
AH4464	LAC* Δ*clfA* Δ*spa*::tetM	this work
AH4743	LAC* Δ*mgrA*::tetM Δ*ebh sraP*::φNΣ Δ*clfA*	this work
MW2	USA400 CA-MRSA	[65]
AH3077	MW2 Δ*arlRS*::erm	this work
AH4330	MW2 Δ*arlRS*::erm φ11::LL29tet *arlRS*	this work
AH3422	MW2 Δ*mgrA*	[22]
AH3491	MW2 Δ*mgrA* φ11::LL29erm *mgrA*	this work
AH3494	MW2 Δ*mgrA* Δ*ebh*::tetL	[22]
AH3945	MW2 Δ*mgrA* Δ*ebh*::tetL Δ*sraP*	[22]
AH3977	MW2 Δ*mgrA* Δ*ebh*::tetL Δ*sraP* Δ*sasG*	[22]
AH4770	MW2 Δ*cna*::tetM	this work
AH4776	MW2 Δ*mgrA* Δ*ebh*::tetL Δ*sraP* Δ*sasG* Δ*cna*::tetM	this work
AH4489	USA100 HA-MRSA, isolate #103	this work
AH5159	#103 Δ*mgrA*::tetM	this work
AH4491	USA100 HA-MRSA, isolate #132	this work
AH5161	#132 Δ*mgrA*::tetM	this work
MRSA252	USA200 HA-MRSA	[66]
AH3483	MRSA252 Δ*mgrA*::tetM	[22]
Newman	MSSA	[67]
AH3472	Newman Δ*mgrA*::tetM	[22]
HG001	MSSA	[68]
AH4546	HG001 Δ*mgrA*::tetM	this work
502A	MSSA	[69]
AH3625	502A Δ*mgrA*::tetM	[22]
*Plasmids*		
pJB38	mutation generation vector, Cam^R^	[70]
pCM29	sGFP expression vector, Cam^R^	[71]
pRMC2	tetracycline-inducible expression vector, Cam^R^	[72]
pALC2073	tetracycline-inducible expression vector, Cam^R^	[72]
pJMB202	arlRS deletion vector, Cam^R^	[23]
pTEV5	His_6_-tagged protein expression vector, Amp^R^	[73]
pEPSA5	xylose-inducible expression vector, Cam^R^	[74]
pHC03	pJB38 Δ*arlRS*::ermB	this work
pHC05	pJB38 *ebh*ΔN	this work
pHC24	pLL29 *arlRS*	this work
pHC30	pHC38 Δ*ebh*1	this work
pHC31	pHC38 Δ*ebh*2	this work
pHC68	mgrA P2-sGFP fusion, Erm^R^	[22]
pHC84	pJB38 Δ*clfA*	this work
pHC87	pRMC2-SasG	this work
pHC88	pRMC2-SasG-His6	this work
pHC89	pALC2073-SasG	this work
pHC90	pALC2073-SasG-His6	this work
pHC187	pEPSA5-*mgrA*	this work
pJK05	pJB38 Δ*cna*	this work
pJK06	pJB38 Δ*cna*::tetM	this work
pJK11	pTEV5-Cna_174-296_	this work

### General reagents

Collagen and 6- and 96-well plates were purchased from Corning, bacteriological growth media and bovine serum albumin (BSA) from Research Products International, and vWF (Humate-P) from CSL Behring. Human Umbilical Vein Endothelial Cells (HUVEC) from pooled donors and cell culture reagents were from Lonza, and the cells were cultured in EGM-2 endothelial growth medium according to manufacturer’s instructions. Biochanin A was from MicroSource Discovery Systems. All other chemicals and proteins, unless noted otherwise, were from Sigma-Aldrich.

### General recombinant DNA and genetic techniques

*Escherichia coli* strains, restriction enzymes, DNAse I, T4 DNA ligase, shrimp alkaline phosphatase, Gibson Assembly Master Mix, and Phusion DNA polymerase were from New England Biolabs (NEB). DNA and RNA isolation kits were from Invitrogen, Qiagen and Ambion. In case of *S*. *aureus*, lysostaphin treatment step was added to the DNA isolation protocol. Plasmids were electroporated into *S*. *aureus* RN4220 as described previously [75]. Bacteriophage transduction between *S*. *aureus* strains was performed with phage 80α or 11 as described previously [76]. Oligonucleotides were ordered from Integrated DNA Technologies; their sequences are listed in the S1 Table. DNA sequencing was performed by the Genomics Division, University of Iowa.

### Plasmid and strain construction

#### Construction of Δ*arlRS*::ermB mutant

To generate the *arlRS*::ermB deletion plasmid pHC03, the erythromycin resistance cassette was amplified from a strain harboring a mariner transposon [77] using primers HC15 and HC16 (S1 Table). The resulting product was digested using NheI and MluI, and ligated into the corresponding sites located between the *arlRS* flanking segments in pJMB202 [23]. This plasmid was electroporated into *S*. *aureus* RN4220, selecting on TSA plates containing Cam at 30 °C. The plasmid was then transduced into *S*. *aureus* strain LAC. Individual colonies were streaked on TSA Cam plates incubated at 42 °C to select for integration into the chromosome. Single colonies were grown in TSB at 30 °C and diluted 1:500 in fresh media for four successive days before diluting to 10^−6^ and plating on TSA containing 0.3 μg/mL anhydrotetracycline to select for loss of the plasmid. Colonies were screened for resistance to Cam and Erm, and Cam^S^ Erm^R^ colonies were screened by PCR for deletion of *arlRS*.

#### Construction of Δ*mgrA*::tetM mutants

The Δ*mgrA*::tetM cassette from AH3455 was transduced into recipient using phage, and deletions were confirmed by PCR with primers HC120 and HC121 (S1 Table) [22].

#### Construction of chromosomal *ebh* truncations

Plasmid pHC05 was constructed to delete the N-terminal domain of Ebh (Δ0.3 MDa, *ebh*ΔN), corresponding to residues 179–2530 in LAC. Regions flanking the deletion were amplified with primers HC28/HC29 and HC30/HC31 and fused using overlap PCR. The resulting fragment was digested with EcoRI and SalI before ligating into pJB38 [70]. Plasmid pHC30 was constructed to delete residues 3659–9450 of Ebh (*ebh*Δ1). Regions flaking the deletion were amplified with primers HC98/HC99 and HC100/HC101 and fused using overlap PCR. The resulting product was digested with SacI and SalI before ligating into pJB38. Plasmid pHC31, to delete residues 6556–9450 of Ebh (*ebh*Δ2), was constructed in a similar fashion, using primers HC104/HC105 and HC100/HC101. Deletions were constructed in LAC as described above for the Δ*arlRS*::ermB mutant, except that colonies were not screened for Erm resistance. Deletions were confirmed by PCR.

#### Construction of Δ*clfA* mutant

Plasmid pHC84 was constructed to generate a marker-less in-frame deletion of *clfA*. Regions upstream and downstream of *clfA* were amplified using primers HC381/HC382 and HC383/HC384, and the pJB38 backbone was amplified using primers HC367/HC368. The resulting fragments were fused by Gibson assembly, and the plasmid was used to generate the chromosomal deletion as described above.

#### Construction of Δ*cna*::tetM mutant

To generate plasmid pJK06 for making a Δ*cna*::tetM mutant, regions upstream and downstream of *clfA* were amplified using primers JK13/JK14 and JK15/JK16, the pJB38 backbone was digested with EcoRI and SalI, the resulting fragments were fused by Gibson assembly, and the resulting plasmid designated pJK05. Afterwards, tetracycline resistance cassette tetM was amplified from the strain AH3455 using primers HC3 and JK18. The resulting product was digested using NheI and KpnI, and ligated into the corresponding sites located between the *cna* flanking segments in pJK05, creating the pJK06 plasmid, which was subsequently used to generate the chromosomal deletion as described above.

#### Construction of the *arlRS* chromosomal complement

To generate pHC24 (pLL29 *arlRS*), a 2398-bp fragment containing the *arlRS* operon and native promoter was amplified using primers HC80 and HC84. The product was digested with BamHI and HindIII and ligated into the same sites in pLL29 [78]. This plasmid was electroporated into RN4220 containing the helper plasmid pLL2787 and integration into the chromosome was confirmed by PCR as described [78]. The integrated construct was then transduced into strain AH1975, selecting for tetracycline resistance.

#### MgrA complementation plasmid

The *mgrA* gene was amplified with primers HC170 and HC171, which add the ribosome binding site for *agrB* in front of the *mgrA* coding sequence. The product was digested with SacI and BamHI before ligating into xylose-inducible vector pEPSA5, to generate pHC187.

#### SasG expression plasmids

The *sasG* gene and its native ribosome binding site were amplified from *S*. *aureus* strain MW2 genomic DNA using primers HC416/HC417. The PCR product was digested with KpnI and SacI, and ligated into pRMC2 [72]. The resulting plasmid, pHC87, expresses *sasG* under the control of an anhydrotetracycline-inducible promoter. The same PCR fragment was also ligated into pALC2073 [79] to generate pHC89, which is anhydrotetracycline inducible but expresses *sasG* at a higher basal level and allows to induce higher level of *sasG* expression. To generate a secreted version of SasG, similar plasmids were constructed that express the first 1338 residues of SasG fused to a C-terminal His6 tag, separated by a single glycine residue. This truncated version of *sasG* was amplified with primers HC416/HC418, digested with KpnI and SacI, and ligated into pRMC2 and pALC2073 to generate pHC88 and pHC90, respectively.

### rSasG purification

*S*. *aureus* LAC expressing a secreted recombinant His_6_-tagged SasG (rSasG) from a pHC90 plasmid was inoculated 1:100 from an overnight culture into 500 ml of tryptic soy broth and grown for 16 h with 100 ng/ml of anhydrotetracycline as an inducer. The spent medium was harvested by centrifugation and filtration through a 0.2 μl filter, and concentrated with a 100 kDA cut-off Ultracell ultrafiltration disc (Millipore) to enrich for the rSasG (≈200 kDa). The protein was further purified on the Ni-charged Bio-Scale Mini Nuvia IMAC Cartridge (Bio-Rad) according to manufacturer’s protocol, dialyzed into PBS, and stored at -80°C.

### SasG gels

To assess the expression of SasG in *S*. *aureus* suspensions used for various assays, bacteria from 1 ml bacteria suspension were lysed by resuspending in 100 μl of a Tris-buffered saline with 0.5 U/ml of lysostaphin and 10 U/ml of DNAse I, and incubating for 1h at 37°C. Cell debris was collected by centrifugation and lysate supernatants were analyzed by SDS-PAGE, stained with silver or Coomassie stains.

### Antibodies against Ebh, ClfA and Cna

The rabbit anti-Ebh serum, and the rabbit polyclonal anti-ClfA antibodies (kindly donated by Joan Geoghegan, Trinity College Dublin) were described previously [23, 80]. Rabbit anti-Cna serum was produced by Pacific Immunology by immunizing rabbits with a recombinant peptide consisting of amino acids 174–296 of the Cna molecule (Cna_174-296_). To clone and purify the Cna_174-296_ peptide, the corresponding sequence was generated by PCR with primers JK79 and JK80 using genomic DNA of *S*. *aureus* MW2 as template, digested with NheI and EcoRI, ligated into pTEV5 to generate pJK11, from which it was expressed as His_6_-N-terminally tagged protein in *E*. *coli* ER2566, and purified on HIS-Select nickel affinity resin (Sigma).

### Clumping assay

Measurement of *S*. *aureus* clumping in the presence of plasma was performed as described previously [22, 23]. Human plasma was added to 1.5 ml of bacterial suspension in PBS to a final concentration of 2.5% v/v, and the mixture was vortexed and incubated at a room temperature for 2h. Clumping, resulting in the sedimentation of clumps, was measured by removing 100 μl aliquot from the top of the tube and measuring its OD_600_. The % of clumping was calculated as the % decrease from the OD_600_ at time 0.

### Static adhesion assay

Cell culture 96-well plates were coated with human fibrinogen, fibronectin, vWF or type I rat collagen by filling with 20 μg/ml protein solution in PBS (or 0.2% acetic acid for collagen) and incubating overnight at 4°C. Afterwards, plates were washed with PBS, blocked by incubating with 5% BSA in PBS for 2h at 37°C, and washed again. Wells were filled with 100 μl of bacterial suspensions in PBS at OD_600_ = 1.0, and incubated for 1h at 37°C. Afterwards, wells were washed and dried, the adherent bacteria were stained with 0.1% crystal violet, the bound stain was solubilized with 33% acetic acid, and the adhesion was measured as the OD_570_ of the resulting solution.

### Flow adhesion assay

Previously described models [10, 81] were adapted to study *S*. *aureus* adhesion under flow conditions. Channels of μ-slides VI^0.4^ (ibiTreat surface, Ibidi) were coated by filling with the protein solutions (as for the static adhesion assay, except that 50μg/ml protein concentrations were used) and incubating overnight at 4°C. Afterwards, channels were washed with PBS, blocked by filling with 5% BSA in PBS for 2h at 37°C, and washed again. Channels were attached to a peristaltic pump (model 205S, Watson-Marlow) and perfused for 10 min with suspensions of GFP-expressing bacteria adjusted to OD_600_ = 0.65 in PBS, at a physiological shear stress of 10 dyn/cm^2^ (shear rate of 1000 s^-1^), calculated as described previously [82]. Afterwards, unbound bacteria were washed away by perfusing with PBS for 10 min, and the remaining attached bacteria were fixed with 2% phosphate-buffered formaldehyde for 15 min. Bacteria adhering to channels were imaged using BZ-X710 fluorescent microscope (Keyence), with images taken of 5 random location in each channel, and subsequently processed using FIJI software [83]: auto thresholding (MaxEnthropy method) was applied to identify and quantify the image area occupied by the adherent bacteria.

Adhesion to endothelial cells under flow was measured in a same way, with HUVECs (at passages 4–6) cultivated in the channels until forming a monolayer, and activated by incubation with 0.1 mM calcium-ionophore A23187 in EBM-2 medium for 10 min at 37°C [10, 81]. Subsequently, the channels were perfused at 37°C for 15 min with *S*. *aureus* suspensions of OD_600_ = 1.0 in DMEM, washed by 10 min perfusion with DMEM, and fixed. When needed, vWF on endothelial surface was visualized by immunostaining as described previously [84], except that secondary antibodies were labelled with Alexa Fluor 568.

### Adhesion to human plasma clots

Human plasma clots were generated in 6-well cell culture plates by mixing 500 μl human plasma with 200 μl of PBS containing 6U/ml thrombin and 0.1 mM CaCl_2_, incubating overnight at 4°C, and washing with PBS, resulting in a layer of clot covering the bottom of the well. Bacterial suspensions in PBS at 5×10^3^ CFU/ml were added to the wells, 2 ml/well. Plates were incubated for 15 min at 37°C in a shaking microplate incubator (Stuart) at 500 rpm, inducing an estimated maximal shear of ≈20 dyn/cm^2^_,_ calculated as described previously [85]. Afterwards, wells were washed extensively with PBS, filled with a melted tryptic soy agar, and adherent CFUs were counted after an overnight incubation at 37°C.

### Unclumping, detachment and clot dissemination assays

#### Unclumping assay

After 2h of clumping of *S*. *aureus* LAC carrying pHC87 plasmid for inducible expression of *sasG* (or control empty plasmid pRMC2), set up in PBS as for the regular clumping assay, the expression was induced by addition of BHI with anhydrotetracycline to the final concentration of 10% v/v and 400 ng/mL, respectively. Tubes were vortexed and incubated at 37°C with shaking. At determined time intervals tubes were removed from the incubator, clumps were allowed to settle for 5 min, OD_600_ of the uppermost 100 μL was measured, and % of clumping calculated in relation to OD_600_ at time 0.

#### Detachment assay

After 1h of attachment to fibrinogen-coated wells and subsequent washing, set up as for a regular adhesion assay, with *S*. *aureus* LAC carrying pHC89 plasmid for inducible expression of *sasG* (or control empty plasmid pALC2073), the expression was induced by filling the wells with 2% BHI in PBS with 400 ng/ml anhydrotetracycline. Plates were placed in a microplate shaker incubator (Stuart), and incubated at 37°C with 500 rpm shaking. At determined time intervals plates were removed from the incubator, wells were washed, amount of adhered bacteria measured using crystal violet staining, and % of adhesion calculated in relation to adhesion at 1h.

#### Dissemination from clot assay

To model infected endovascular vegetation, human infected clots were generated *ex vivo* by mixing 215 μL human plasma with 85 μL of PBS containing 6 U/mL thrombin, 0.1 mM CaCl_2_, and 2.4×10^9^ CFU/ml of *S*. *aureus* LAC carrying pHC89 plasmid for inducible expression of *sasG* (or control empty plasmid pALC2073), and allowing it to coagulate for 15 min at 37°C. Afterwards, the infected clots were washed extensively with PBS, placed in a 6-well cell culture plates filled with 2 mL of 10% BHI in PBS with 400 ng/mL anhydrotetracycline, and incubated at 37°C in a shaking microplate incubator (Stuart) at 500 rpm, inducing an estimated maximal shear of ≈20 dyn/cm^2^. At determined time intervals, the numbers of CFU disseminating from the infected clot into the surrounding medium were measured by plating the medium on a tryptic soy agar (TSA).

### Immunofluorescence microscopy of *S*. *aureus*

Immunofluorescence microscopy was used to visualize various proteins on *S*. *aureus* surface. Bacteria in PBS were allowed to attach to Superfrost Plus microscope slides (Fisher), and afterwards were fixed for 15 min with 2% formaldehyde solution, blocked with 5% BSA in PBS for 1 h, stained with primary antibody at 4°C overnight and secondary Alexa488-conjugated goat anti-rabbit IgG antibody for 1h (all antibodies diluted in 5% BSA in PBS), mounted with Fluoroshield Mounting Medium with DAPI (Abcam), and observed with BZ-X710 fluorescent microscope. All LAC strains used for imaging lacked protein A (Δ*spa* or *spa*::φNΣ). In case of MW2 strains, 1% human serum was added to blocking solution and antibody solutions to saturate protein A and block unspecific binding.

### Western blots

Western blot of proteins sheared from *S*. *aureus* surface with anti-Ebh serum was described previously [22]. For Western blot of cell-wall anchored proteins, the cell wall fraction was prepared as described previously [86] and stained with anti-ClfA or anti-Cna antibodies analogously as for the anti-Ebh staining. All LAC strains used lacked protein A (Δ*spa*, Δ*spa*::tetM, or *spa*::φNΣ). In case of MW2 strains, 1% human serum was added to all solutions to block unspecific binding to protein A.

### Measurement of surface properties of *S*. *aureus*

The relative surface hydrophobicity was measured using the microbial adhesion to hydrocarbon (hexadecane) assay, as described previously [87], except that addition of ammonium sulfate (needed for the gram-negative bacteria in the original protocol) was omitted. The relative surface charge was measured by electrostatic interaction chromatography on a Dowex 1×8 anion exchange resin, 100–200 mesh, as described previously [88].

### Single-cell force spectroscopy

Fibrinogen was immobilized on substrates via self-assembled monolayers of thiols. Practically, gold-coated glass coverslips were immersed overnight in an ethanol solution containing 1 mM of 10% 16-mercaptododecahexanoic acid/90% 1-mercapto-1-undecanol, rinsed with ethanol, and dried with nitrogen. They were then immersed for 30 min into a solution containing 10 mg×ml^-1^ N-hydroxysuccinimide (NHS) and 25 mg×ml^-1^ 1-ethyl-3-(3-dimethylaminopropyl)-carbodiimide (EDC), rinsed with ultrapure water (ELGA LabWater), incubated with 0.5 mg×ml^-1^ human fibrinogen for 1 h, rinsed further with PBS buffer, and immediately used without de-wetting. The abiotic substrates were obtained by immersing gold-coated glass coverslips overnight in an ethanol solution containing 1 mM of dodecanethiol (Sigma) for CH_3_ substrates, 1mM of 1-mercapto-1-undecanol (Sigma) for OH substrates or 1 mM of 16-mercaptododecahexanoic acid (Sigma) for COO^-^ substrates. These surfaces were rinsed in ethanol and dried with nitrogen before to use.

Bacterial cell probes were obtained as previously described [89, 90]. Briefly, colloidal probes were obtained by attaching single silica microsphere (6.1 μm diameter, Bangs laboratories) on triangular shaped tipless cantilevers (NP-O10, Microlevers, Bruker Corporation) with UV-curable glue (NOA 63, Norland Edmund Optics) using a Nanoscope VIII multimode AFM (Bruker Corporation, Santa Barbara, USA). These colloidal probes were then incubated for 1 h in a 10 mM TBS (pH 8.5) containing 4 mg×ml^-1^ of dopamine hydrochloride (99%). They were then rinsed in TBS and directly used for cell probe preparation. The nominal spring constant of the colloidal probe cantilever was ~0.06 N×m^-1^ as determined by the thermal noise method. 50 μl of diluted bacterial suspension in exponential phase was deposited into a petri dish containing fibrinogen-coated or abiotic surfaces at a distinct location, and filled with 3 ml of PBS. Single bacterium was attached on the center of the colloidal probes using a Bioscope Catalyst AFM (Bruker Corporation, Santa Barbara, USA) equipped with a Zeiss Z1 Axio Observer and a model C10600 Hamamatsu camera. Cell probes were used to measure cell–substrates interaction forces at room temperature (20°C), using an applied force of 250 pN, a constant approach-retraction speed of 1 μm×s^-1^ and a contact time of 100 ms. Data were analyzed using the Nanoscope software from Bruker (Santa Barbara, USA). Adhesion forces were obtained by calculating the maximum adhesion force for each curve. The results from independent measurements were merged. For each condition, experiments were repeated for at least 3 times with independent cultures.

### Single-molecule force spectroscopy

Gold cantilevers (OMCL-TR4, Olympus Ltd., Tokyo, Japan) with a nominal spring constant of ~0.02 N×m^-1^ were functionalized with fibrinogen using thiols chemistry as previously described for substrates. The spring constants of the cantilevers were measured using the thermal noise method. Bacteria from exponential phase culture were immobilized by mechanical trapping into porous polycarbonate membranes (Millipore, Billerica, USA) with a pore diameter of 0.8 μm. After filtering a cell suspension, the membrane was rinsed with PBS, cut into piece (1 x 1 cm^2^) and attached to a steel sample puck using a small piece of double-face adhesive tape. The mounted sample was transferred into the AFM liquid cell while avoiding de-wetting. Bare tips were first used to localize and image individual cells and then replaced by functionalized tips. Adhesion maps were obtained by recording 32-by-32 force-distance curves on areas of 500 x 500 nm^2^, using an applied force of 250 pN, a constant approach-retraction speed of 1 μm×s^-1^ and a contact time of 100 or 500 ms. Data were analyzed using the Nanoscope software from Bruker (Santa Barbara, USA). Adhesion forces were calculated considering the last peak for each curve and adhesive events are displayed as light pixels. For each condition, experiments were repeated for at least 3 times with independent cultures.

### Intravital microscopy of *S*. *aureus* adhesion to injured carotid arteries

Previously described intravital microscopy method was used [91, 92]. Female C57BL/6 mice, 8–11 week old, were anesthetized with katamine/xylazine, and GFP-expressing bacteria (2.2×10^8^ CFU/mouse in 50 μl PBS) were injected intravenously through the retro-orbital plexus. The common carotid artery was exposed and 1×2 mm piece of Whatman paper saturated with 10% FeCl_3_ was applied to it to induce endothelial injury and thrombosis. Afterwards the field was washed with PBS, and thrombus formation and *S*. *aureus* attachment were monitored with a Nikon upright microscope equipped with a high-speed electron-multiplying camera. Images were collected at determined time points, and the percent of the artery area occupied by the adherent fluorescent *S*. *aureus* was were calculated using FIJI image analysis software [83].

### Intravital microscopy of *S*. *aureus* adhesion to mesenteric circulation

A previously described intravital microscopy method was used [10, 81]. Female 7-week old C57BL/6 mice were anesthetized with a ketamine/xylazine. Their peritoneal cavity was opened via midline abdominal incision, and their mesenteric circulation was exposed. To activate mesenteric endothelium, 5 μl of a 10 mM Ca^2+^-ionophore A23187 was topically applied to the surface of the mesentery. Afterwards, GFP-expressing bacteria (1.2×10^8^ CFU/mouse in 50 μl PBS) were injected intravenously through the retro-orbital plexus. After 30 minutes, all visible adhesion of *S*. *aureus* to mesenteric vasculature was assessed and captured with a Nikon upright microscope with a digital camera. If no adhesion was observed, representative images of vessels were captured.

### Screen for ArlRS inhibitors

A previously described reporter of ArlRS activity—that is *S*. *aureus* USA300 LAC strain carrying reporter plasmid pHC68 in which expression of sGFP is driven by the P2 promoter of *mgrA* (P2 promoter is entirely dependent on arlRS activity for its expression, and it accounts for ≈90% of all *mgrA* transcripts in the cell), thus allowing for measurement of GFP fluorescence as the proxy for ArlRS activity in bacteria growing in a presence of tested compounds [22] was used to screen the “Spectrum Collection” library (MicroSource Discovery Systems), consisting of 2320 small molecules, for potential ArlRS inhibitors. The reporter strain was grown in 96-well plates in TSB with erythromycin in the presence of 50 μM of each compound. Plates were grown at 37 °C with shaking in a Stuart incubator, and growth (OD_600_) and fluorescence were monitored over 24 h. Potential hits were further evaluated by growing *S*. *aureus* USA300 LAC Δ*spa* in TSB with 100 μM compounds for 24h, and assaying level of expressed Ebh in culture supernatants by the dot-blot, as described previously [22].

### Biochanin A and inhibition of ArlRS signaling experiments

Biochanin A used for experiments were dissolved in DMSO as a vehicle to create stock solutions. To determine antibacterial activity of biochanin, its MIC was measured with broth microdilution method as detailed by Clinical and Laboratory Standards Institute (CLSI) M07-A10 guidelines, except that BHI was used as a growth medium. To study the effect of biochanin A on *S*. *aureus* growth, overnight cultures of *S*. *aureus* were diluted 100× in fresh BHI supplemented with biochanin A (or DMSO vehicle control at the same volume) at 40 μM and 80 μM, incubated with shaking at 37°C, and OD_600_ of the culture measured at regular time intervals. For all the other experiments, *S*. *aureus* was grown as for other assays, to an OD_600_ = 1.5, but biochanin A (or DMSO vehicle control at the same volume) was added to the medium to a final concentration of 40 μM or 80 μM. Afterwards, bacteria were used either for the RNA isolation and qPCR, for the clumping assay, or for the static fibrinogen adhesion assay as described above. For experiments investigating mechanism of action of biochanin A, additional *S*. *aureus* strains were used: LAC strain lacking giant surface proteins (Δ*ebh sraP*::φNΣ), and LAC strains expressing mgrA from a xylose-inducible plasmid pHC187 (or empty control plasmid pEPSA5) grown in presence of 1% xylose.

### RNA isolation and qPCR

Bacteria were washed with ice-cold PBS, and RNA was purified with commercial kits as described before [22]. To perform a quantitative PCR (qPCR), cDNA was generated with the High Capacity cDNA Reverse transcription Kit (Applied Biosystems). Primers JK45 and JK46 were used for *mgrA*, and 41995031X and 419950330X for DNA gyrase (*gyrB*), as described previously [22], with primer efficiencies of 97% and 88%, respectively. qPCR was performed by amplifying 20 ng of cDNA in 20 μl total reaction volume with iTaq Universal SYBR Green Supermix (Bio-Rad) in CFX96 Touch Real-Time PCR System (Bio-Rad) under the following conditions: 3 min at 95°C, 40 cycles of 15 s at 95°C and 30 s at 55°C, followed by a dissociation curve. No template and no reverse transcription controls were performed in parallel. Data were analysed and Cq were determined with CFX Manager 3.1 (Bio-Rad). Expression was normalized to that of *gyrB*, and values represent three biological replicates.

### Statistical analysis

For all assays data were pooled from at least two independent experiments. Differences between *S*. *aureus* strains were analyzed by ANOVA with a Dunnett’s multiple comparisons post-test. In case of strains expressing increasing levels of SasG, increasingly truncated Ebh, or exposed to increasing concentration of ArlRS inhibitor, the differences were analyzed by ANOVA with a post-test for linear trend. All CFU data were normalized by log transformation before statistical analysis. Two-tailed *p* values were calculated. Prism 7 (GraphPad Software) was used for statistical calculations.

## Supporting information

S1 Table Oligonucleotides used in this study. (DOCX)

S1 Fig ArlRS–MgrA cascade controls adhesion to endovascular ligands through regulation of surface proteins in *S*. *aureus* MW2. Adhesion of *S*. *aureus* strains MW2 and its respective mutants lacking elements of the regulatory cascade ArlRS–MgrA to fibrinogen (**A**), fibronectin (**B**), and vWF (**C**) was tested. Adhesion of *S*. *aureus* strains MW2 with the *mgrA* deletion alone or with additional deletions of the giant surface proteins regulated by *mgrA* to fibrinogen (**D**), fibronectin (**E**), and vWF (**F**) was tested. All adhesions assays were performed in a static 96-well plate assay. N = 6 per group. *p<0.05, **p<0.01, ***p<0.001, ****p<0.0001, compared to WT. Data presented as mean ± SEM.
(TIF)

S2 Fig Conventional adhesins are still present on surface of the ArlRS–MgrA cascade mutants. Adhesion of *S*. *aureus* strains with mutations in *mgrA* and in various surface adhesins on LAC background to fibrinogen (**A**) and on MW2 background to collagen (**B**) was tested in a static 96-well plate assay. N = 6 per group. ****p<0.0001, compared to WT. Data presented as mean ± SEM. Additionally, presence of ClfA fibrinogen adhesin on cell surface of LAC (**C, E**) and of Cna collagen adhesin on surface of MW2 (**D, F**) was demonstrated with western blot of cell wall fractions of the cells (**C-D**) and with immunofluorescence microscopy (**E-F**).
(TIF)

S3 Fig Adhesion defect in ArlRS–MgrA cascade mutants is not due to a changed surface hydrophobicity or charge. Relative surface hydrophobicity (**A**) and relative negative surface charge (**B**) of *S*. *aureus* LAC strain and its mutant derivatives were measured. N = 6 per group. ****p<0.0001, compared to WT. Data presented as mean ± SEM.
(TIF)

S4 Fig Addition of anhydrotetracycline inducer has no effect on clumping and adhesion if SasG-expressing vector is absent. Clumping (**A**) and adhesion to fibrinogen in 96-well plates (**B**) of *S*. *aureus* LAC carrying empty Tet-inducible expression vectors pRMC2 (**A**) and pALC2073 (**B**) was measured after addition of anhydrotetracycline (aTet) to the growth medium, or after addition of soluble rSasG to bacterial suspensions. Amount of SasG expressed by *S*. *aureus* was measured by SDS-PAGE, stained with silver (**C**) or Coomassie stain (**D**), and representative images out of two independent experiments are shown. N = 6 per group, no significant differences observed. Data presented as mean ± SEM.
(TIF)

S5 Fig Truncated variants of the Ebh protein are present on the cell surface of *S*. *aureus mgrA* mutants. A series of chromosomal deletions in the *ebh* was constructed, resulting in the expression of progressively shorter Ebh proteins in the LAC Δ*mgrA* (**A**). Their presence on the *S*. *aureus* cell surface was demonstrated with western blot of sheared cell surface proteins (**B**) and with immunofluorescence microscopy (**C**).
(TIF)

S6 Fig Induction of *sasG* expression is necessary for its effect. No effect on clumping (**A**), adhesion to fibrinogen in 96-well plates (**B**), and dissemination from an infected plasma clot (**C**) of *S*. *aureus* LAC carrying Tet-inducible SasG expression vectors pRMC2-SasG (**A**) and pALC2073-SasG (**B-C**), was observed in absence of anhydrotetracycline induction. N = 6 per group. Data presented as mean ± SEM.
(TIF)

S7 Fig *S*. *aureus* binds to endothelial cells both directly and indirectly through vWF. Images of GFP-expressing *S*. *aureus* adhering to endothelial cell monolayers. vWF multimers secreted by the cells were stained with immunohistochemistry and are labeled red with Alexa Fluor 568. *S*. *aureus* can be seen predominantly adhering to the strings of vWF multimers (arrows), although couple bacteria adhere also directly to the monolayer independent from vWF (arrowheads). Two representative microscopy images from two independent experiments are shown (image size: 450 μm × 450 μm).
(TIF)

S8 Fig Biochanin A has moderate effect on *S*. *aureus* growth. Growth of *S*. *aureus* strain LAC in BHI supplemented with different doses of biochanin A (or equal volume of DMSO solvent) was recorded as OD_600_. N = 2. Data presented as mean ± SEM.
(TIF)
