# Supplementary material for: Staphylococcus aureus adhesion in endovascular infections is controlled by the ArlRS–MgrA signaling cascade
Source: PLoS Pathog. 2019 May 22;15(5):e1007800. doi: 10.1371/journal.ppat.1007800 (PMC6548404; doi:10.1371/journal.ppat.1007800)
Supplement: S1 Table — (DOCX) [file ppat.1007800.s001.docx]

**S1 Table. Oligonucleotides used in this study**

| **Code** | **Name** | **Sequence** |
| --- | --- | --- |
| HC3 | TetM 5’NheI | GTTAGCTAGCCCTAGGCAAATATGCTCTTACGTGC |
| HC15 | ErmB 5’NheI | GTCA GCTAGC AGGAGGGATTCGTCATGTTG |
| HC16 | ErmB 3’MluI | GTCA ACGCGT AAAAAGCAGGAAAATGAAATGG |
| HC28 | EbhN delA EcoRI | GCATGAATTCGCGAAATTGTATTGAATCGTCA |
| HC29 | EbhN delB | CTTTTTACCTTCAGTGCTTACTGGATGTTGTACGTTACCATTATCAGATG |
| HC30 | EbhN delC | CATCTGATAATGGTAACGTACAACATCCAGTAAGCACTGAAGGTAAAAAG |
| HC31 | EbhN delD SalI | GTTCGTCGACAACGCAGATTGCACTTCTTG |
| HC80 | ArlS down 3’HindIII | CCC AAGCTT GGGAGTACAACAGAAATGATAAAGAACCACTG |
| HC84 | ArlR prom 5’BamHI | CCC GGATCC ATTGCGGTAAGGCCTTGTGTTACAG |
| HC98 | GArepeat del1A SacI | GAC GAGCTC CAAGCTGTTGCAGATAAAGATCAAG |
| HC99 | GArepeat del1B | CTCGAGGGTACCGCTAGCTTGATCTAATGTATTGGCATTTGATTGAAC |
| HC100 | GArepeat del1C | GCTAGCGGTACCCTCGAGGCAATGGAAACATTGAAACATTTAGTTGAC |
| HC101 | GArepeat del1D SalI | CGT GTCGAC TCAACATCTTGTTTCGCATCTTCTT |
| HC104 | GArepeat del2A SacI | GAC GAGCTC GACAATGCAACGACAGTAGCAG |
| HC105 | GArepeat del2B | CTCGAGGGTACCGCTAGCATTATTAAGTGATGTAGCACTATTTTTCAGATCAG |
| HC120 | MgrA upstream | TGCACAAATCATACCTAATTGCTT |
| HC121 | MgrA downstream | AGGCGAAAAGATTGCGATT |
| HC170 | MgrA 5’SacI + RBS | GTTGAGCTCCAGTGAGGAGAGTGGTGTAAAAATGTCTGATCAACATAATTTAAAAGAAC |
| HC171 | MgrA 3’BamHI | GTTGGATCCTTATTTTTCCTTTGTTTCATCAAATGCATG |
| HC367 | pJB38 rev Gibson | CGG GTA CCG AGC TCG AAT TCT TG |
| HC368 | pJB38 fwd Gibson | GGG ATC CTC TAG AGT CGA CCT GC |
| HC381 | clfA delA | CTTTCGTCTTCAAGAATTCGAGCTCGGTACCCGGAA TCA GAG CTA GTA GAC CAA ATT CG |
| HC382 | clfA delB | TGC TTC ATC TTC CTC GAG ACG CGT GCT AGC CTT CAT ATT CAT TTT ATT CCC TCT TTT TA |
| HC383 | clfA delC | AAAATGAATATGAAGGCTAGCACGCGTCTCGAGGAAGATGAAGCAAATACGTCACTAA |
| HC384 | clfA delD | CAT GCC TGC AGG TCG ACT CTA GAG GAT CCC AGA ACT CCT CAC TTT GTT TTT TCA AAG |
| HC416 | MW2 sasG 5’ KpnI | GTTGGTAC CCACTGTAAGTAAAGTGGAAAATATGGAA |
| HC417 | MW2 sasG 3’ SacI | GTT GAGCTC  TAG GTC TTT CAA TCC AAC TTT TGG |
| HC418 | MW2 sasG His 3’SacI | GTT GAG CTC TTA ATG ATG ATG ATG ATG ATG ACC TTC TGC TCG TTT TTT CTC TTG AT |
| HC441 | pJB38 rev2 Gibson | GAG CTC GAA TTC TTG AAG ACG AAA GG |
| HC442 | pJB38 fwd2 Gibson | GTCGACCTGCAGGCATGC |
| JK13 | cna delA | GAG GCC CTT TCG TCT TCA AGA ATT CGA GCT CGA CGA TAG AAT ATG CTA AGC C |
| JK14 | cna delB | GAG TTC TCG AGG GTA CCG TCG GTA CCG CTA GCC AAC ACG TTC TTG TTC ATA A |
| JK15 | cna delC | GCT AGC GGT ACC GAC GGT ACC CTC GAG AAC TCA TAA ACC ATT ATA ATT ATT TTT ATA G |
| JK16 | cna delD | GTG AAA TCA GAG CTT GCA TGC CTG CAG GTC GAC TGA TGA GGA TTT ACA CCT AGC |
| JK18 | TetM KpnI R | GCA TGG TAC CGC ACT AAG TTA TTT TAT TGA ACA TAT ATC TTA C |
| JK45 | mgrA_qPCR_F1 | CTA ACA TAC CCA CAA TTT CTT GTC T |
| JK46 | mgrA_qPCR_R1 | CTG TAC CAG TAT CGA GTG CTA AT |
| JK79 | Cna174-296_F_NheI | CGAGCTAGCTATTATAAAACGGGAGATATG |
| JK80 | Cna174-296_R_EcoRI | GCTAGAATTCTTGTGAATTATTAACAAACTC |
| 41995031X | SA-gyrBFor | AAC GGA CGT GGT ATC CCA GTT GAT |
| 41995030X | SA-gyrBRev | CCG CCA AAT TTA CCA CCA GCA TGT |
