# Supplementary figures and images for: Staphylococcus aureus adhesion in endovascular infections is controlled by the ArlRS–MgrA signaling cascade
Source: PLoS Pathog. 2019 May 22;15(5):e1007800. doi: 10.1371/journal.ppat.1007800 (PMC6548404; doi:10.1371/journal.ppat.1007800)

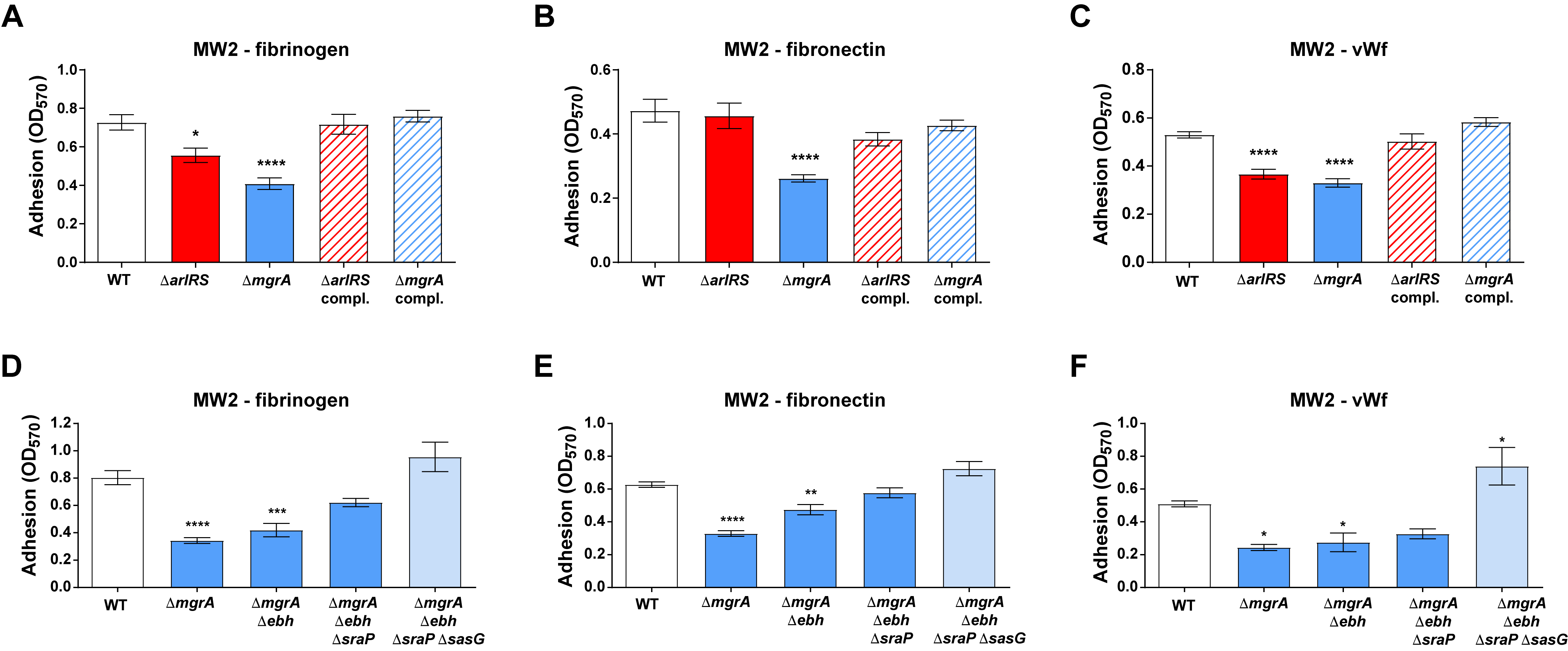

Supplement: S1 Fig — Adhesion of S. aureus strains MW2 and its respective mutants lacking elements of the regulatory cascade ArlRS–MgrA to fibrinogen (A), fibronectin (B), and vWF (C) was tested. Adhesion of S. aureus strains MW2 with the mgrA deletion alone or with additional deletions of the giant surface proteins regulated by mgrA to fibrinogen (D), fibronectin (E), and vWF (F) was tested. All adhesions assays were performed in a static 96-well plate assay. N = 6 per group. *p<0.05, **p<0.01, ***p<0.001, ****p<0.0001, compared to WT. Data presented as mean ± SEM. (TIF) [file ppat.1007800.s002.tif]

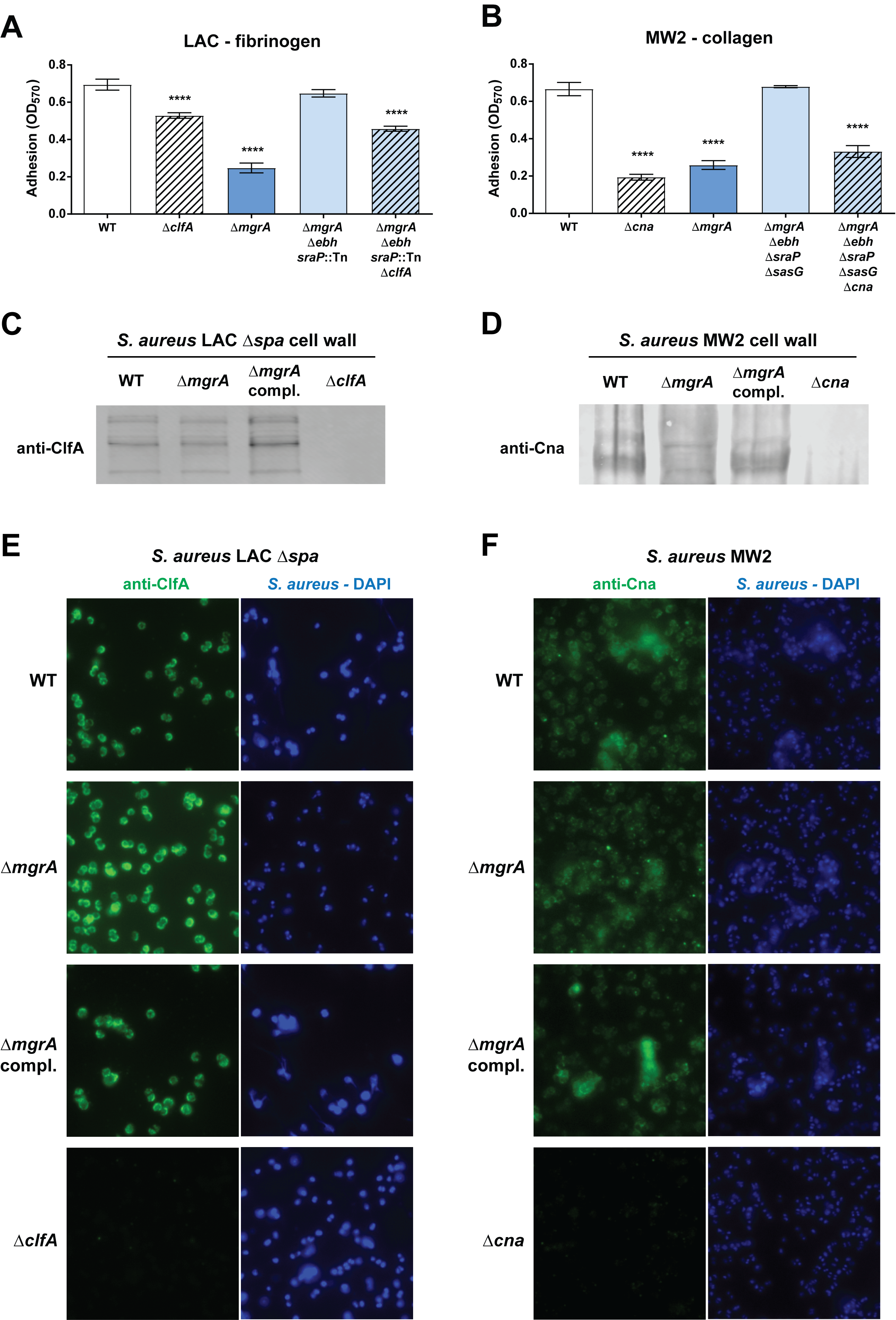

Supplement: S2 Fig — Adhesion of S. aureus strains with mutations in mgrA and in various surface adhesins on LAC background to fibrinogen (A) and on MW2 background to collagen (B) was tested in a static 96-well plate assay. N = 6 per group. ****p<0.0001, compared to WT. Data presented as mean ± SEM. Additionally, presence of ClfA fibrinogen adhesin on cell surface of LAC (C, E) and of Cna collagen adhesin on surface of MW2 (D, F) was demonstrated with western blot of cell wall fractions of the cells (C-D) and with immunofluorescence microscopy (E-F). (TIF) [file ppat.1007800.s003.tif]

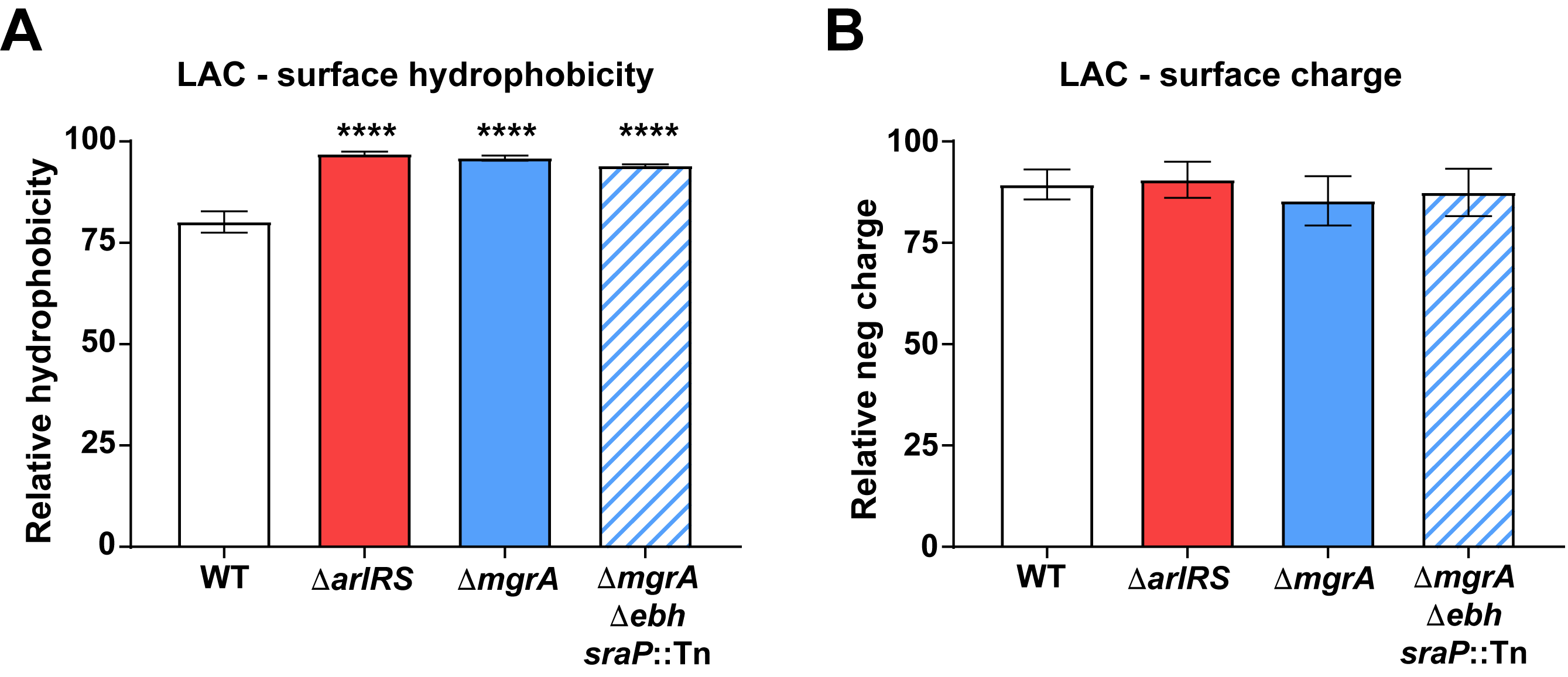

Supplement: S3 Fig — Relative surface hydrophobicity (A) and relative negative surface charge (B) of S. aureus LAC strain and its mutant derivatives were measured. N = 6 per group. ****p<0.0001, compared to WT. Data presented as mean ± SEM. (TIF) [file ppat.1007800.s004.tif]

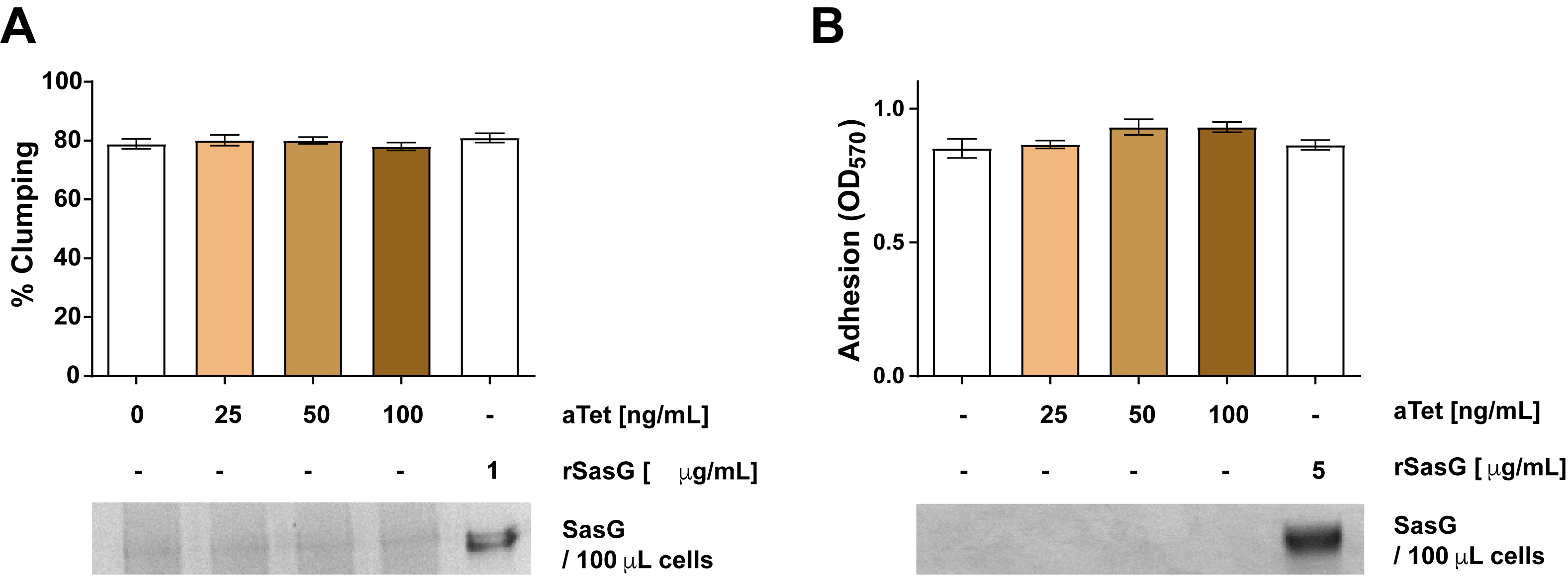

Supplement: S4 Fig — Clumping (A) and adhesion to fibrinogen in 96-well plates (B) of S. aureus LAC carrying empty Tet-inducible expression vectors pRMC2 (A) and pALC2073 (B) was measured after addition of anhydrotetracycline (aTet) to the growth medium, or after addition of soluble rSasG to bacterial suspensions. Amount of SasG expressed by S. aureus was measured by SDS-PAGE, stained with silver (C) or Coomassie stain (D), and representative images out of two independent experiments are shown. N = 6 per group, no significant differences observed. Data presented as mean ± SEM. (TIF) [file ppat.1007800.s005.tif]

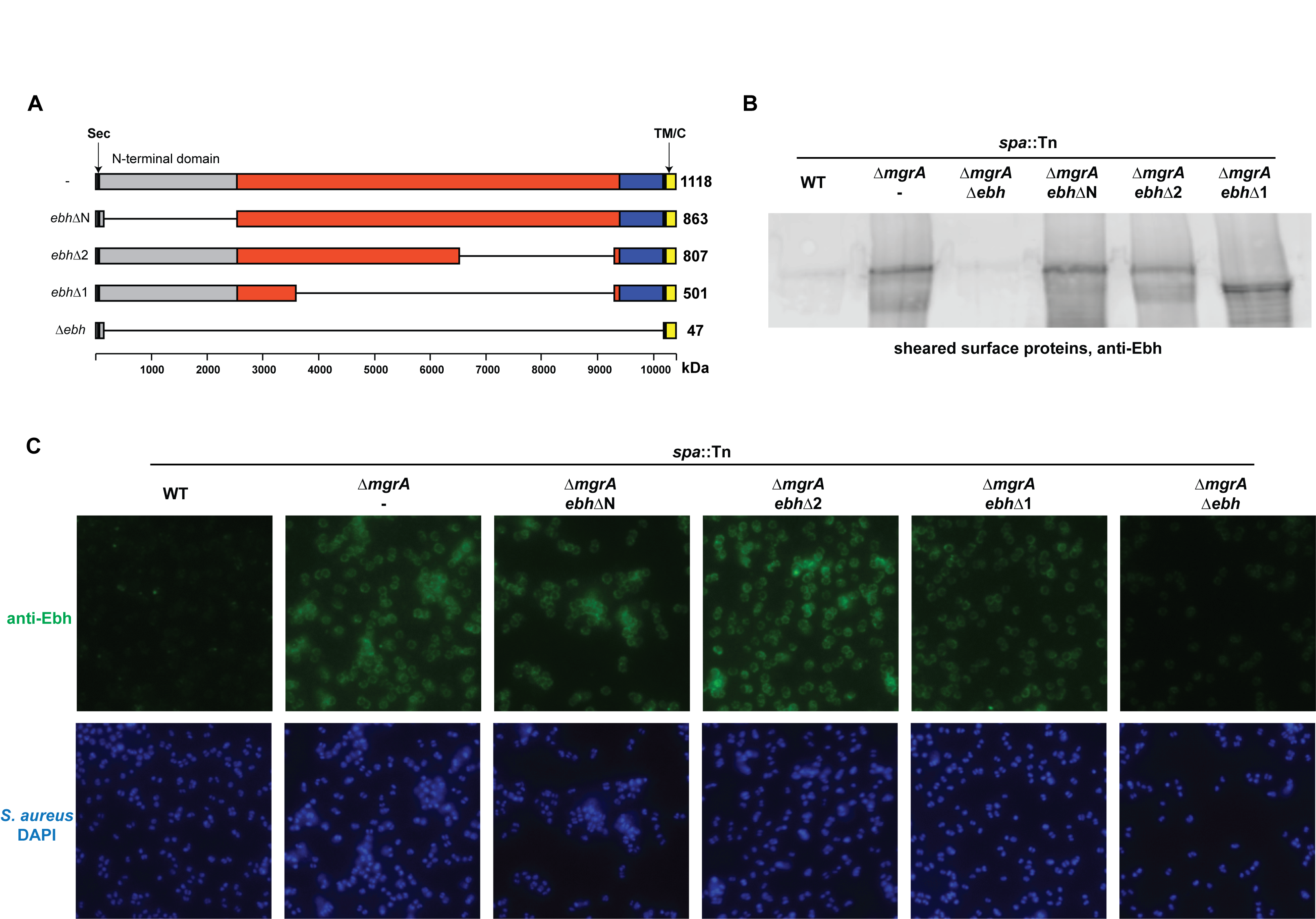

Supplement: S5 Fig — A series of chromosomal deletions in the ebh was constructed, resulting in the expression of progressively shorter Ebh proteins in the LAC ΔmgrA (A). Their presence on the S. aureus cell surface was demonstrated with western blot of sheared cell surface proteins (B) and with immunofluorescence microscopy (C). (TIF) [file ppat.1007800.s006.tif]

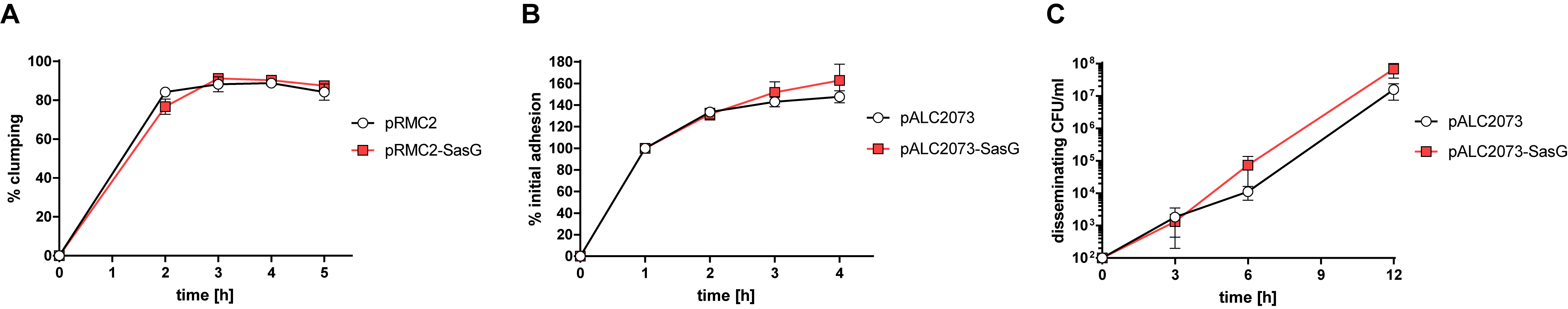

Supplement: S6 Fig — No effect on clumping (A), adhesion to fibrinogen in 96-well plates (B), and dissemination from an infected plasma clot (C) of S. aureus LAC carrying Tet-inducible SasG expression vectors pRMC2-SasG (A) and pALC2073-SasG (B-C), was observed in absence of anhydrotetracycline induction. N = 6 per group. Data presented as mean ± SEM. (TIF) [file ppat.1007800.s007.tif]

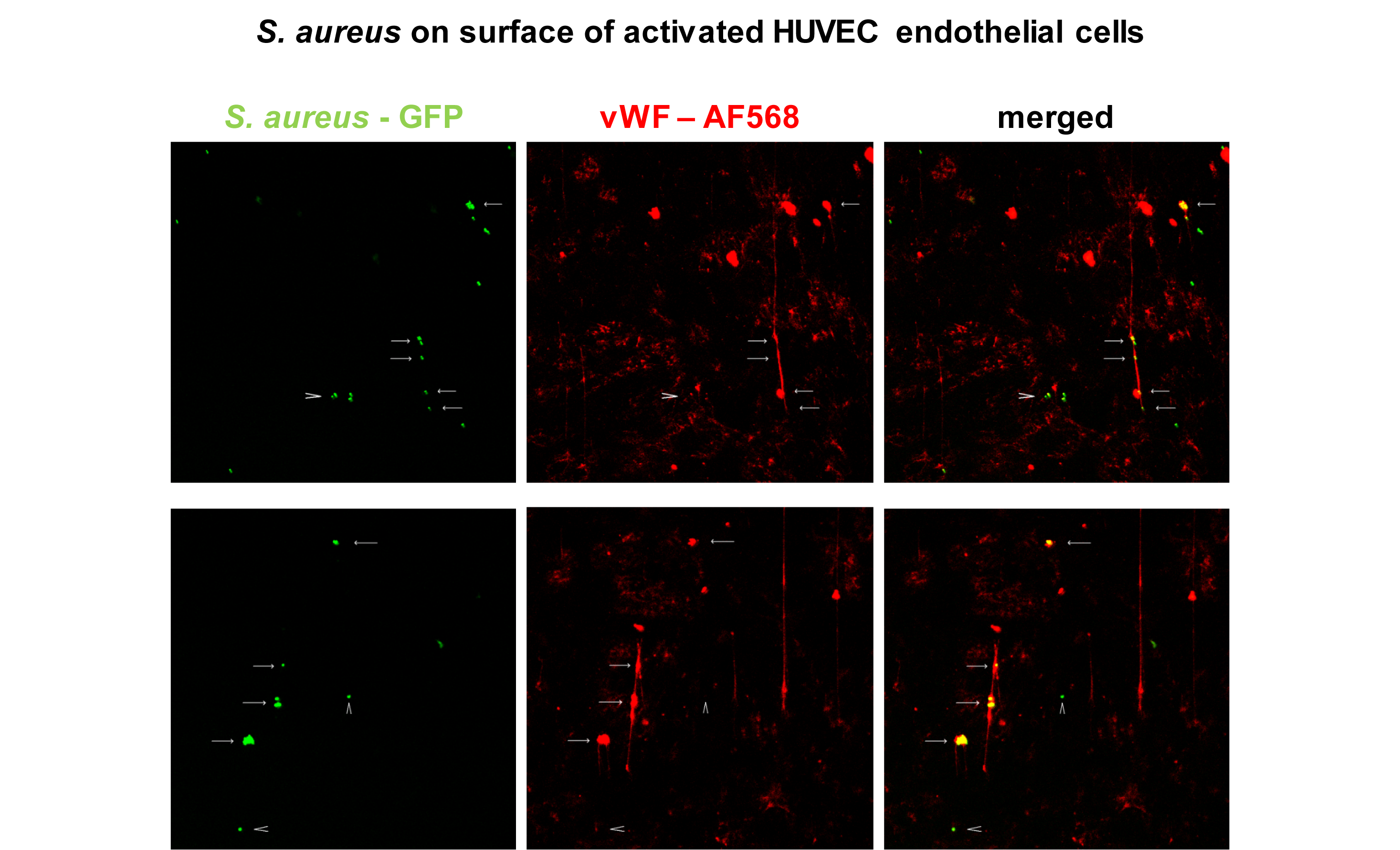

Supplement: S7 Fig — Images of GFP-expressing S. aureus adhering to endothelial cell monolayers. vWF multimers secreted by the cells were stained with immunohistochemistry and are labeled red with Alexa Fluor 568. S. aureus can be seen predominantly adhering to the strings of vWF multimers (arrows), although couple bacteria adhere also directly to the monolayer independent from vWF (arrowheads). Two representative microscopy images from two independent experiments are shown (image size: 450 μm × 450 μm). (TIF) [file ppat.1007800.s008.tif]

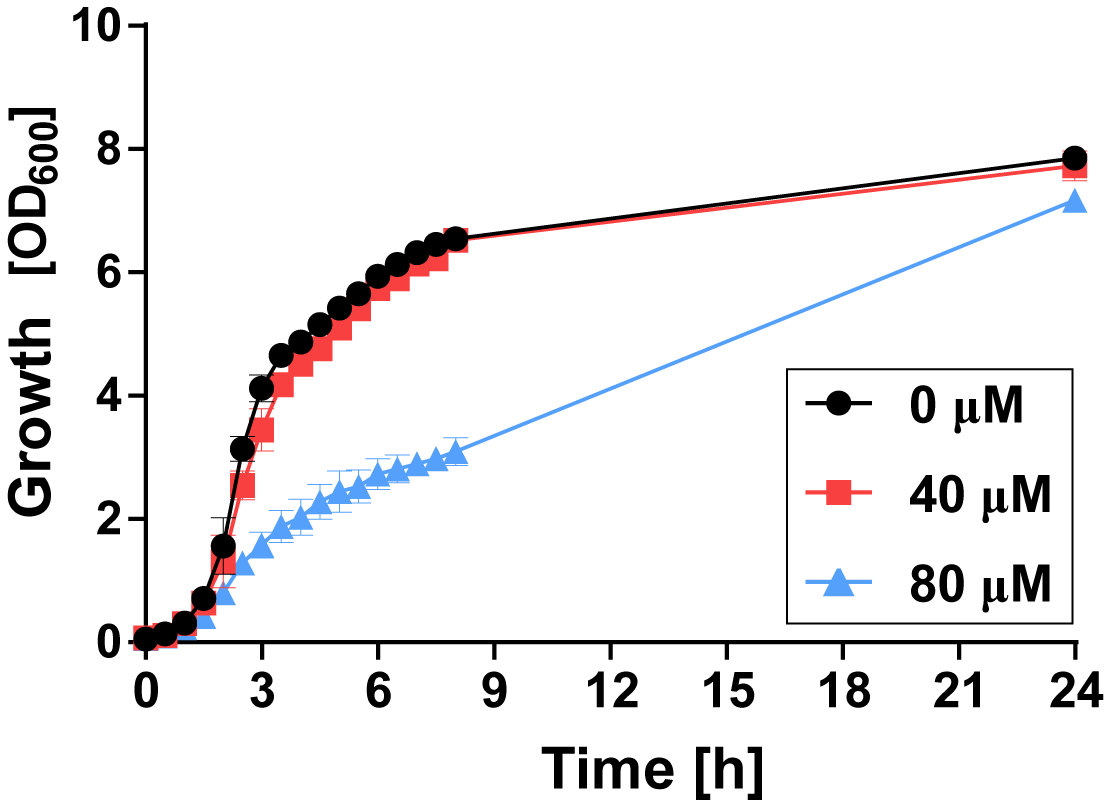

Supplement: S8 Fig — Growth of S. aureus strain LAC in BHI supplemented with different doses of biochanin A (or equal volume of DMSO solvent) was recorded as OD600. N = 2. Data presented as mean ± SEM. (TIF) [file ppat.1007800.s009.tif]
